# MicroRNA172b-5p/trehalose-6-phosphate synthase module stimulates trehalose synthesis and microRNA172b-3p/AP2-like module accelerates flowering in barley upon drought stress

**DOI:** 10.3389/fpls.2023.1124785

**Published:** 2023-03-06

**Authors:** Aleksandra Swida-Barteczka, Andrzej Pacak, Katarzyna Kruszka, Przemyslaw Nuc, Wojciech M. Karlowski, Artur Jarmolowski, Zofia Szweykowska-Kulinska

**Affiliations:** ^1^ Department of Gene Expression, Institute of Molecular Biology and Biotechnology, Faculty of Biology, Adam Mickiewicz University, Poznań, Poland; ^2^ Department of Computational Biology, Institute of Molecular Biology and Biotechnology, Faculty of Biology, Adam Mickiewicz University, Poznań, Poland

**Keywords:** barley, drought, MicroRNAs, flowering, trehalose-6-phosphate synthase, APETALA2, osmoprotection

## Abstract

MicroRNAs (miRNAs) are major regulators of gene expression during plant development under normal and stress conditions. In this study, we analyzed the expression of 150 conserved miRNAs during drought stress applied to barley ready to flower. The dynamics of miRNAs expression was also observed after rewatering. Target messenger RNA (mRNAs) were experimentally identified for all but two analyzed miRNAs, and 41 of the targets were not reported before. Drought stress applied to barley induced accelerated flowering coordinated by a pair of two differently expressed miRNAs originating from a single precursor: hvu-miR172b-3p and hvu-miR172b-5p. Increased expression of miRNA172b-3p during drought leads to the downregulation of four APETALA2(AP2)-like genes by their mRNA cleavage. In parallel, the downregulation of the miRNA172b-5p level results in an increased level of a newly identified target, trehalose-6-phosphate synthase, a key enzyme in the trehalose biosynthesis pathway. Therefore, drought-treated plants have higher trehalose content, a known osmoprotectant, whose level is rapidly dropping after watering. In addition, trehalose-6-phosphate, an intermediate of the trehalose synthesis pathway, is known to induce flowering. The hvu-miRNA172b-5p/trehalose-6-phosphate synthase and hvu-miRNA172b-3p/AP2-like create a module leading to osmoprotection and accelerated flowering induction during drought.

## 1 Introduction

MicroRNAs (miRNAs) are a class of small RNA(sRNA) molecules whose length varies between 20 and 22 nt. The major function of miRNAs is the posttranscriptional downregulation of gene expression during plant development and stress adaptation (Bologna et al., 2018). MiRNAs, in most cases, are imbedded within independent transcriptional units—*MIRs*, which are transcribed by RNA polymerase II (RNA Pol II) (Xie et al., 2005). MiRNA expression regulation is an essential mechanism allowing plants for adaptation to adverse environmental conditions. One of the major abiotic stresses worldwide is drought, and it is a main cause of grain yield loss (Helman and Bonfil, 2022). Barley is fourth on the list of cereals produced; therefore, several attempts have been made to explore for barley miRNAs that are connected with the drought response. Drought in barley induces many changes at the level of miRNAs. The number of observed deregulated miRNAs depends strongly on the developmental stage, susceptibility, or tolerance of a studied cultivar to water shortages, or the duration and strength of the applied stress. So far, the most comprehensive study on drought-deregulated barley miRNAs revealed 25 downregulated, conserved miRNAs and one upregulated in a 3-week-old Golden Promise cultivar (Hackenberg et al., 2015). A comparison between two barley cultivars, drought-sensitive Morocco 9e75 and drought-resistant Yousef treated with prolonged water shortage stress starting from four-leaf developmental stage, revealed different responses at the level of miRNA expression. Interestingly, miRNAs, mostly novel, were upregulated in drought-resistant Yousef while the sensitive cultivar Morocco 9e75 responded with the downregulation of the miRNAs (Fard et al, 2017). The diverse expression of four conserved miRNAs and one novel miRNA was observed in the leaves of four different barley cultivars exposed to prolonged drought starting at the 3-week-old plants and lasting until flag leaves’ appearance: Commander, Fleet, Hindmarsh, and WI4304. These cultivars are characterized by similar fitness in drought conditions (Ferdous et al., 2017). Such discrepancies between cultivars were also observed at the level of pri-miRNA expression. Drought affected the levels of 44 pri-miRNAs in the cultivar Sebastian, while the same conditions altered the expression of 84 pri-miRNAs in Rolap (Swida-Barteczka et al., 2016). Drought-deregulated miRNAs are a good putative target to improve plant stress tolerance. For example, novel barley miRNA, hvu-x13-downregulated during severe drought, induced drought tolerance when overexpressed in barley (Zhou et al., 2018; Smoczynska et al., 2020).

One of the well-recognized roles of miRNAs in plants is flowering regulation by miRNA172. The vegetative growth of plants is characterized by a high expression of miRNA156, which downregulates a group of SQUAMOSA promoter binding protein–like (SBP-like/SPL)/SQUAMOSA promoter binding protein (SBP) transcription factors (TFs). A high level of SPLs inhibits adventitious root formation, shoot branching, and leaf formation (Xu et al., 2016). Therefore, the high level of miRNA156 during the early stages of plant development allows for biomass accumulation. The switch between the dominant levels of miRNA156 and miRNA172 marks the end of the vegetative growth stage and the beginning of the generative growth stage. Highly expressed miRNA172 downregulates the level of APETALA2-like (AP2-like) family member TFs by translational inhibition. AP2-like TFs are responsible for the inhibition of flowering initiation and flower patterning (Aukerman and Sakai, 2003; Chen, 2004). In maize, the miRNA172-driven downregulation of GLOSSY15 (GL15), an orthologue of AP2-like TF, promotes the transition from juvenile to adult growth visible as not only reproductive organ development but also changes in leaf morphology (Lauter et al., 2005).

MiRNA and miRNA* are diced out of the pre-miRNA stem together as an miRNA/miRNA* duplex (Papp et al., 2003; Kurihara and Watanabe, 2004). MiRNAs* are usually quickly degraded and present at low levels (Kruszka et al., 2013; Smoczynska et al., 2020). However, there are numerous studies presenting a high accumulation of miRNA* under specific growth conditions or in particular organs. MiRNA408b* and miRNA528* are accumulated in barley shoots under nitrogen excess. Nevertheless, as their targets are not identified, their biological role is not revealed (Grabowska et al., 2020). The proportion of a given miRNA and its’ partner miRNA* also changes as was shown during grain filling in *Oryza sativa* for four miRNA/miRNA* pairs. These are miRNA1425/miRNA1425*, miRNA1433/miRNA1433*, miRNA1884b/miRNA1884b*, and miRNA408/miRNA408*. Importantly, the targets for three miRNAs*: miRNA1425*, miRNA1433*, and miRNA1884b*, were identified (Peng et al., 2013). The differences between miRNA and miRNA* accumulation were also reported in the case of six miRNA/miRNAs* during *Orchis italica* flowering (Aceto et al., 2014).

Trehalose biosynthesis is an essential part of plant metabolism. Particularly, it was found that it acts as an osmoprotectant during drought (Figueroa and Lunn, 2016). The rate of trehalose biosynthesis and utilization is a sensor of the metabolic status of the cell. Therefore, the abolition of trehalose biosynthesis has detrimental effects to plants. The knockout mutant of *Arabidopsis thaliana* TREHALOSE-6-PHOSPHATE SYNTHASE 1 (TPS1)—*tps1* is characterized by a lowered frequency of cell divisions and altered sugar, lipid, and protein accumulation at the embryonic stage of development that, together, leads to embryo lethality (Gómez et al., 2006). Increased trehalose biosynthesis improves plant productivity and tolerance to various abiotic stresses including drought. For example, rice plants overexpressing bifunctional fusion genes consisting of bacterial TPS (*otsA*) and bacterial trehalose-6-phosphate phosphatase (*otsB*) genes have an increased catalytic efficiency of trehalose synthesis. The increased supply of trehalose is linked with a higher soluble carbohydrate level and an elevated capacity of photosynthesis (Garg et al., 2002). Similarly, such positive results of trehalose overproduction were also visible in *Nicotiana tabacum* and Arabidopsis. In these plants, *Saccharomyces cerevisiae* genes encoding TPS1 (*ScTPS1*) and TPP (*ScTPS2*) were fused and expressed under a drought-inductive promoter. The transgenic tobacco and Arabidopsis plants were characterized by increased turgor and higher water retention when exposed to drought, while the initial relative water content was the same as in wild-type plants (Karim et al., 2007).

The results of this study present a comprehensive study of barley drought- and rehydration-responsive conserved miRNAs as well as their targets. Moreover, they show that flowering timing and osmoprotective trehalose synthesis are regulated by miRNA 172b-3p, its functional miRNA* (172b-5p), and their targets—AP2-like and TPS, respectively.

## 2 Materials and methods

### 2.1 Plant material

**Figure 1:**
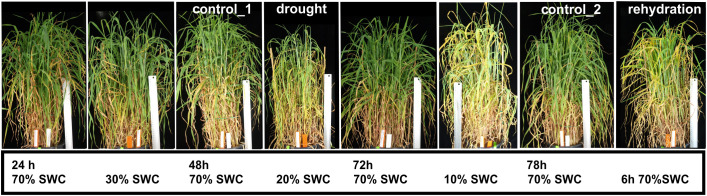
Barley (*Hordeum vulgare*, genotype Rolap) plants’ appearance during the course of a drought and rehydration stress experiment. Drought stress was applied by withholding watering, after 48 h, when soil water content (SWC) dropped to 20%, plants were collected and treated as drought-stressed. Next day, when SWC reached 10%, plants were watered, collected 6 h later and treated as rehydrated. Control_1 (C1) and control_2 (C2) plants were collected for drought (D)- and rehydration (R)-treated samples, respectively.

The spring barley seeds of the genotype Rolap (a doubled haploid line derived from Roland and Apex cultivars) were obtained from the Institute of Plant Genetics of the Polish Academy of Sciences in Poznan, Poland (Devaux et al., 1992). Plants were cultivated in pots, in autoclaved field soil mixed with sand (7:2), supplemented with multinutrients and later with a straight nitrogen fertilizer. In a Conviron (Winnipeg, Manitoba, Canada) growth chamber, 22°C day/15°C night temperatures, a 16 h day/8 h night photoperiod, and 800 µmol light conditions were maintained. Plants were grown under a controlled watering regime, and a soil water content (SWC) of 70% was considered to be optimal. Flag leaf appearance, stage 39–41 of the Zadoks cereal development decimal code, was the beginning of drought/rewatering stress treatment (Zadoks et al., 1974). Drought stress was applied by the withholding of water (described in detail in Swida-Barteczka et al., 2016). Shoots were collected 48 h later when SWC dropped to 20%; the next day, the remaining plants were rewatered to 70% of SWC and collected after 6 h (**Figure 1**). The shoots of four plants treated with one of the two stress conditions were pooled together and treated as one biological replica. Three biological replicas were tested. For each stressed sample, the shoots of four control plants were collected. For caryopsis measurements, 20% SWC was maintained for 5 days to allow self-fertilization and caryopsis development. For rehydration stress caryopsis measurements, after 48 h of 20% SWC drought, the plants were rewatered; optimal watering was maintained for 3 days. After that time, spikes emerging from main shoots were collected and fixed. Spikes were fixed in formaldehyde:ethanol:acetic acid (10%:50%:5%) solution for 24 h in 4°C and stored in 70% ethanol; before the measurements, the spikes were rehydrated. Caryopses were dissected from fixed shoots and flowers, and their width was measured. Caryopses for the analysis were chosen counting from the bottom of the spike; the two lowest flowers were not taken to analysis. No more than five caryopses were taken from one spike.

### 2.2 RNA isolation

Total RNA was isolated with a method that allows for the enrichment of a small RNA fraction. The method was described in detail elsewhere (Kruszka et al., 2013). Shortly, RNA was extracted from 100 mg plant powder with 38% phenol saturated with 0.1 M sodium acetate supplemented with 0.8 M guanidine thiocyanate, 0.4 M ammonium thiocyanate, 0.1 M sodium acetate, 5% glycerol, 0.5% sodium lauroylsarcosine, and 5 mM 2,2',2'',2'''-(Ethane-1,2-diyldinitrilo)tetraacetic acid (EDTA). Polysaccharides were removed with Ambion Plant RNA Isolation Aid (Thermo Fisher Scientific, Waltham, MA, USA). RNA was precipitated with 1 volume of 0.8 M sodium citrate in a 1.2 M sodium chloride solution and 1 volume of isopropanol. The RNA quality and quantity were measured with a NanoDrop ND-1000 (Thermo Scientific) spectrophotometer. RNA integrity was estimated on agarose gel. For small RNA library preparation, RNA quality was verified with an Agilent 2100 Bioanalyzer and Nano Plant RNA assay (Agilent)/RNA 6000 Nano Assay. The RNA integrity number (RIN) values of the tested samples were consistently higher than 7.5.

### 2.3 Northern blot analysis of mature microRNAs

Denaturing 8M urea polyacrylamide gel electrophoresis (PAGE) (15%) was used to separate 90 µg of total RNA. The separated RNA was transferred to Amersham Hybond-NX nitrocellulose (GE Healthcare) and UV-crosslinked. MicroRNAs were visualized with DNA oligo probes (Sigma) 5′ labeled with [γ-32P]ATP (6000 Ci mmol^–1^; Hartmann Analytic GmbH). A U6 small nuclear RNA (snRNA) hybridization signal was used as a loading control. The blots were exposed to a phosphor imaging screen (Fujifilm), scanned with a Fujifilm FLA5100 reader (Fujifilm Co., Ltd), and quantified with Multi Gauge V2.2 software (Tokyo, Japan). Probes are listed in **Supplementary Table 1**.

### 2.4 Quantitative real-time RT-PCR

Total RNA was treated with Ambion TURBO DNase (Thermo Fisher Scientific) to remove contaminating DNA. cDNA was reverse-transcribed from 3 µg of DNA-depleted RNA using Invitrogen SuperScript III Reverse Transcriptase (Thermo Fisher Scientific) and 0.5 µg Oligo(dT)18 Primer (Thermo Fisher Scientific). Reverse transcription quantitative real-time PCR (RT-qPCR) was run on 7900HT Fast Real-Time PCR System (Applied Biosystems). Reactions were set with Power SYBR Green PCR Master MIX (Applied Biosystems), two mRNA-specific primers (a final concentration of 200 nM each), and 1 µl of a fourfold diluted template. The final reaction volume was 10 µl. RT-qPCR reaction was performed for three biological replicas. Primers are listed in **Supplementary Table 1**.

### 2.5 Small RNA library preparation and analysis

Small RNA libraries were prepared independently for the three biological replicas of drought- stressed, rewatered, and control plants; altogether, 12 libraries were constructed. The libraries were set with an Illumina TruSeq Small RNA Library Preparation Kit (Illumina, San Diego, CA, USA) and quantified using a Quant-iT PicoGreen dsDNA Assay kit (Molecular Probes, Thermo Fisher Scientific, Waltham, MA, USA) reagent and Tecan Infinity 200 Pro Spectrometer (Tecan, Männedorf, Switzerland). Next-generation sequencing (NGS) was run on a HiScan SQ machine (Illumina). The details of the library construction procedure were described elsewhere (Pacak et al., 2016). NGS data were analyzed as described in detail previously (Alaba et al., 2015). Sequences with 10 counts in at least one of the 12 libraries were analyzed further. To identify conserved microRNA, sequencing reads were mapped to the miRBase (20 and 22) database (Kozomara and Griffiths-Jones, 2014) using a blastn-short algorithm. The mapping results were further filtered to include matches with no substitutions; longer or shorter reads were accepted. Isomirs were manually removed from further analysis.

### 2.6 Degradome library preparation and target prediction

The degradome preparation by the parallel analysis of RNA ends (PARE) method and analysis were described in detail in another publication (German et al., 2009; Alaba et al., 2015). Shortly, poly(A)-enriched RNA was used to prepare 26–27 nt cDNA tags derived from the 5’ ends of RNA. RNA and DNA adaptors were designed to fit to the TruSeq sequencing system of Illumina. Two libraries were constructed, one from drought stressed (as) and another from control (ak) plants. Libraries were sequenced in Fasteris SA (Plan-les-Ouates, Switzerland). Reads with identified adaptors and at least 14 nt long after adaptor trimming were subsequently analyzed as described in Alaba et al. (2015). RNA-Seq results were processed using cutadapt version 1.8.1 (http://dx.doi.org/10.14806/ej.17.1.200) to remove low-quality reads and adapter sequences. The trimmed reads were mapped to the full mRNA sequences downloaded from the National Center for Biotechnology Information (NCBI) nucleotide database with bowtie version 1.1.0 (Langmead et al., 2009) and the following parameters: bowtie -q -v 0 —best -a -S. The mapped reads were processed to score only the 5’ end coordinates and presented as T-plots. Optionally, read counts were normalized to the average signal detected on the whole transcript. cDNAs are labeled as Morex locus (MLOCs); the MLOC numbers and sequences [downloaded from Ensembl Plants, version 31 (Kersey et al., 2014)] identified as target mRNAs are listed in **Supplementary Table 5**.

### 2.7 5’ RACE

To examine the cleavage sites of predicted target mRNAs, 5' rapid amplification of cDNA ends (5′RACE) experiments were conducted with the GeneRacer Kit (Invitrogen) according to the manufacturer’s protocol with modifications: RNA dephosphorylation and decapping steps were omitted. PCR products were cloned into the pGEM T-Easy vector (Promega) and sequenced (Faculty’s Laboratory of Molecular Biology Techniques, Adam Mickiewicz University, Poznań, Poland).

### 2.8 Western blot

Proteins were extracted from 100 mg of main spike tissue immediately after sampling. Sampled tissue was ground on ice in 3 volumes of a protein extraction buffer (50 mM Tris-HCl, pH 7.5, 100 mM NaCl, 0.25% TritonX-100, 1mM EDTA, 10 mM NaF, 1 mM Na_3_VO_3_, 0.25% NP-40, 1 mM PMSF, 1 × protease inhibitor EDTA-free (Roche Diagnostics GmbH, Mannheim, Germany), 10 µM MG132), centrifuged and frozen in the presence of loading buffer (0.35 M Tris-HCl (pH 6.8), 10.28% (w/v) SDS, 36% (v/v) glycerol, 5% beta-mercaptoethanol, 0.012% (w/v) bromophenol blue). Protein concentration was measured with a Bradford assay (Bradford Reagent, Bio-Rad) and 40 µg of proteins were run on 12% sodium dodecyl sulfate–polyacrylamide gel electrophoresis (SDS-PAGE) gel. Proteins were transferred to an Immobilon-P transfer membrane (Millipore) with Trans-Blot SD Semi-Dry Transfer Cell (Bio-Rad, Hercules, CA, USA) for 1 h. The membrane was blocked in 3% milk in phosphate-buffered saline (PBS). Proteins were detected and visualized with a PHY0960S Agrisera antibody (1:1,000), an AS:09602 Goat anti-Rabbit IgG (H&L) Agrisera secondary antibody (1:25,000), and Amersham ECL (Cytiva) reagents.

### 2.9 Trehalose and abscissic acid content measurement

Trehalose content was assessed with the anthrone method as described by Li et al. (2014) with changes. Shortly, 100 mg of grinded tissue was extracted for 1 h with 80% (v/v) hot ethanol. The supernatants were dried in 80°C and dissolved in 250 µl of water, and 100 µl of this extract was used for trehalose content measurement. Moreover, 150 µl of 0.2 N H_2_SO_4_ was added to the assay extract, boiled for 10 min, and chilled on ice. Then, 150 µl of 0.6 N NaOH was added to the said mixture, boiled for 10 min, and chilled on ice. Next, 2 ml of the anthrone reagent (0.05 g anthrone in 100 ml of 95% H_2_SO_4_) was added, the assay mixture was boiled for 10 min and chilled, and the absorbance was measured at 630 nm. The trehalose concentration was calculated using a standard curve and stated as mg g^-1^ fresh weight (FW).

Abscissic acid (ABA) content was measured with ultrapressure liquid chromatography tandem mass spectrometry (UHPLC-MS/MS, Shimadzu Nexera XR UHPLC/LCMS-8045 system; Kyoto, Japan) on an Ascentis Express C-18 column (2.7 µm, 100 × 2.1 mm, Sulpeco, Bellefonte, PA, USA). The detailed procedure of ABA extraction and measurement is described in Smoczynska et al. (2022).

### 2.10 Statistical analysis

The differential level of miRNA accumulation was estimated using the DESeq2 package in R (Love et al., 2014). The method fits negative binomial generalized linear models for each gene, uses the Wald test for significance testing, and reports the Benjamini–Hochberg-adjusted p-value. The miRNA expression pattern was presented as log_2_ (fold change) for sequences with p ≤ 0.05. MiRNAs with log_2_(fold change) ≥ 0.6 or ≤ -0.6 were considered as differentially expressed and further analyzed. RT-qPCR data were analyzed with Sequence Detection System, version 2.4.1 (Applied Biosystems) and Microsoft Excel 2016. The R^2^ values of the data (≥0.997) were calculated with LinRegPCR software (Ramakers et al., 2003). Results were standardized to the barley ADP-ribosylation factor 1-like [GenBank: AJ508228.2] internal reference (Rapacz et al., 2012). Expression levels were calculated with the relative quantification method (2^-ΔΔCt^) and presented as FC (Dolata et al., 2021). The FC values between 0 and 1 were recalculated (=-1/FC) to show them as –FC. The significance of the changes was tested with a Student’s *t*-test.

### 2.11 Bioinformatics

Bioinformatical analyses also included BLASTN comparisons against NCBI (http://ncbi.nlm.nih.gov) and International Barley Blast Server (https://webblast.ipk-gatersleben.de/barley_ibsc/) databases (Johnson et al., 2008). Target genes were identified by the alignment of the identified target MLOC sequence to Morex v2 Gene Models (2019) (Home (ipk-gatersleben.de)) (Colmsee et al., 2015). Primers were designed with the help of Primer3web version 4.1.0 software (https://bioinfo.ut.ee/primer3/) (Untergasser et al., 2012). The Pri-miRNA folding: Folder version 1.11 (RNAfold algorithm) software is available at http://www.ncrnalab.dk/#rnafolder/rnafolder.php.

### 2.12 Accession numbers

All data are deposited in the NCBI Sequence Read Archive database under project number PRJNA526135 and accession numbers SRR8698942, SRR8698943, SRR8698944, SRR8698945, SRR8698946, SRR8698960, SRR8698961, SRR8698962, SRR8698963, SRR8698964, SRR8698965, SRR8698966, SRR8698967, and SRR8698968.

## 3 Results

### 3.1 microRNA level during drought and rehydration undergoes rapid changes

Spring barley plants *Hordeum vulgare*, genotype Rolap were cultivated in pots under controlled temperature, light, and watering regimes. Drought stress was applied by withholding watering when a flag leaf appeared in 50% of the plants. Plants were collected when SWC dropped to 20% (**Figure 1**). Drought-treated plants were rewatered the next day and collected 6 h later. Control plants were collected in parallel for drought (control_1)- and rehydration (control_2)-treated plants, respectively. All treatments and controls were sampled in three biological repeats, giving together 12 samples (3 × control_1, 3 × drought, 3 × control_2, 3 × rehydration).

To identify conserved miRNAs, sRNA-enriched total RNA was isolated and Illumina TruSeq Small RNA libraries were constructed independently for each treatment and biological repetition. The 12 libraries were sequenced on a HiScan SQ system (Illumina). The predominant fractions of sRNA reads were 24 and 21 nt long molecules (**Supplementary Figure 1**). Sequences with at least 10 reads in one or more of the 12 libraries were mapped to plant miRNAs from the miRBase (20 and 22) database with no substitutions allowed but length differences accepted. Isomirs were removed manually from further analysis. Altogether, 150 conserved barley miRNAs were identified (**Table 1**; **Supplementary Table 2**). The sequences of identified conserved barley miRNAs, their names, and miRBase annotated sequences are listed in **Supplementary Table 2**. The mean expression level of each conserved barley miRNA was calculated as the number of reads from six sRNA libraries, C1 and D for drought or C2 and R for the rehydration experiment, respectively.

**Table 1:** Summary of changes of conserved microRNA (miRNA) expression in drought- or rehydration-treated barley .

miRNA expression	↑	↓	=
drought	11	38	101
rehydration	9	16	125
common	5	11	92

↑ – number of upregulated, ↓ – number of downregulated, = – number of not changed miRNAs; common – number of miRNAs upregulated, downregulated, or unchanged together in both treatments. The full list of conserved miRNA reads is presented in **Supplementary Table 2**.

Conserved barley miRNAs differentially expressed during drought or rehydration were identified with DESeq package in R. The miRNAs whose expression exceeds mean counts (p ≤ 0.05) are considered to be differentially expressed, and the change is presented as log_2_ (fold change) (**Supplementary Table 2**). Drought elevates the level of 11 conserved miRNAs out of the 150 identified, while the level of 38 miRNAs is decreased (**Table 1**). During rehydration, the expression of 9 miRNAs is upregulated, and the expression of 16 miRNAs is downregulated. Interestingly, rewatering restores the level of 6 out of 11 drought-upregulated miRNAs and 27 out of 38 drought-downregulated miRNAs within 6 h. The level of 101 and 125 of the conserved miRNAs remains unchanged, respectively, in drought and rehydration (**Table 2**; **Supplementary Table 2**).

**Table 2:** MicroRNAs downregulated or upregulated in drought and/or rehydration .

microRNA	expression change [log_2_ FC]			
	Drought	p-value	Rehydration	p-value
gma-miR4995	2.59	***	2.92	***
ath-miR8175	2.36	***	1.89	***
gma-miR6300	2.10	***	1.98	***
ptc-miR6478	1.45	***	1.12	***
bdi-miR5054	1.03	**	1.68	***
gma-miR5368	2.02	***	1.06	
osa-miR5072	1.38	*	0.69	
bna-miR167d	1.37	*	0.34	
hvu-miR172b-3p	1.07	*	0.26	
hvu-miR156a/b	1.01	*	0.43	
ppt-miR894	0.73	*	0.23	
hvu-miR5051	-0.73	*	-2.06	***
ata-miR171a-3p	-0.93	*	-1.11	*
ata-miR168-3p	-0.96	***	-1.21	***
bdi-miR166e-3p	-1.81	***	-1.00	*
bdi-miR827-5p	-1.85	***	-1.01	*
hvu-miR171-5p	-2.20	***	-0.69	*
ata-miR166c-5p	-2.32	***	-1.96	***
ata-miR166a-5p	-2.45	***	-0.93	**
ata-miR1432-5p	-2.45	***	-2.06	***
ata-miR5168-5p	-4.20	***	-2.07	***
ata-miR396c-3p	-4.58	***	-1.66	***
aly-miR396b-5p	-0.63	*	-0.56	
cme-miR166g	-0.65	*	0.06	
ata-miR166c-3p	-0.73	*	0.01	
ata-miR5168-3p	-0.82	**	-0.29	
gma-miR156k	-0.86	**	-0.41	
hvu-miR168-5p	-0.87	**	-0.29	
csi-miR166d	-0.87	**	-0.24	
aqc-miR166c	-0.87	*	-0.28	
bdi-miR159a-3p	-0.90	*	0.09	
hvu-miR159a/b	-1.12	***	-0.30	
ata-miR398f-3p	-1.14	*	-0.49	
ata-miR166d-5p	-1.17	**	-0.06	
hvu-miR6196	-1.22	*	-0.54	
osa-miR319a-3p.2-3p	-1.53	***	-0.23	
ata-miR167c-3p	-1.58	**	0.10	
ata-miR171c-5p	-1.60	**	0.50	
aly-miR166a-5p	-1.60	***	-0.86	
osa-miR166e-3p	-1.79	*	0.20	
bdi-miR159b-5p.1	-1,93	***	-0.87	
aly-miR399b-3p	-1.93	***	-0.58	
hvu-miR172b-5p	-2.00	***	-0.40	
ata-miR166e-5p	-2.03	*	-0.56	
bdi-miR159b-5p.3	-2.24	**	0.25	
bdi-miR164a-3p	-2.38	**	-0.47	
hvu-miR5049f	-2.41	*	-0.53	
ata-miR395a-3p	-2.54	***	0.48	
ata-miR408-5p	-4.20	***	-0.93	
aly-miR164a-5p	0.12		1.88	***
bdi-miR156h-3p	1.11		1.48	*
cme-miR156j	0.79		0.87	*
ahy-miR159	0.65		0.65	*
zma-miR168a-3p	0.03		-0.86	**
bdi-miR1432	-0.30		-0.95	**
tae-miR9773	-0.64		-1.04	**
ata-miR156c-3p	-1.25		-1.12	*
bdi-miR408-3p	-0.28		-1.51	*

The up- and downregulated miRNAs were based on log_2_ fold change (≥ ± 0.6) values, and their significance is based on the p-value (***p≤0.001, **p≤0.01, *p≤0.05). Expression changes are presented as log_2_ fold change. Up- and downregulated miRNAs are marked as red and blue, respectively.

**Figure 2:**
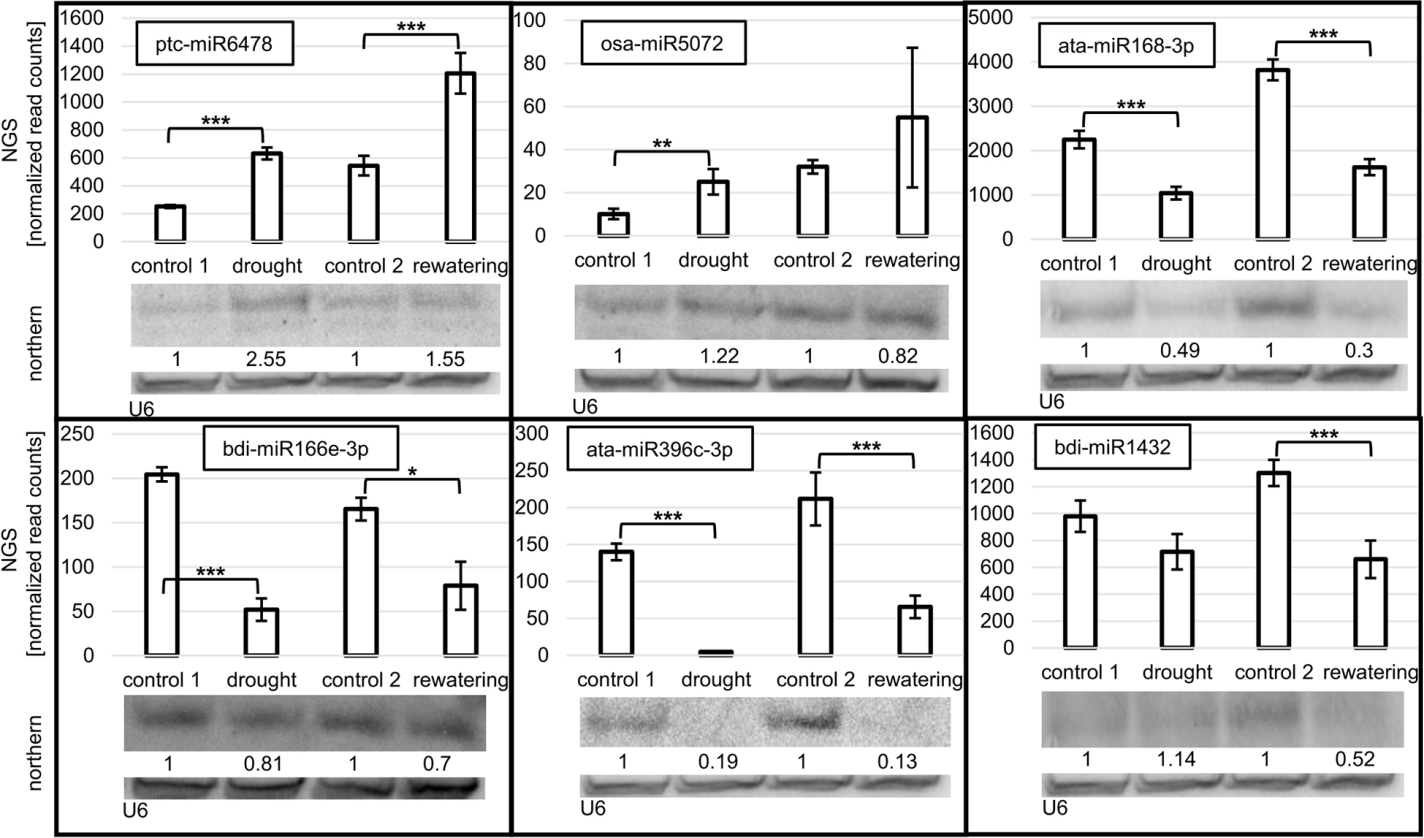
Accumulation of selected miRNAs in drought and rehydration established by northern and compared to next-generation sequencing (NGS) normalized read counts. The significance of the observed NGS differences was calculated with the DESeq package in R (***p ≤ 0.001, **p ≤ 0.01, and *p ≤ 0.05). Numbers below blot images are relative intensities of the miRNA bands, Control signals in each blot were treated as 1. The microRNA hybridization signal was corrected with U6 as a loading control. MiRNAs for northern analysis were selected to confirm the different accumulation profiles of various miRNAs during drought and rehydration revealed by NGS.

**Figure 3:**
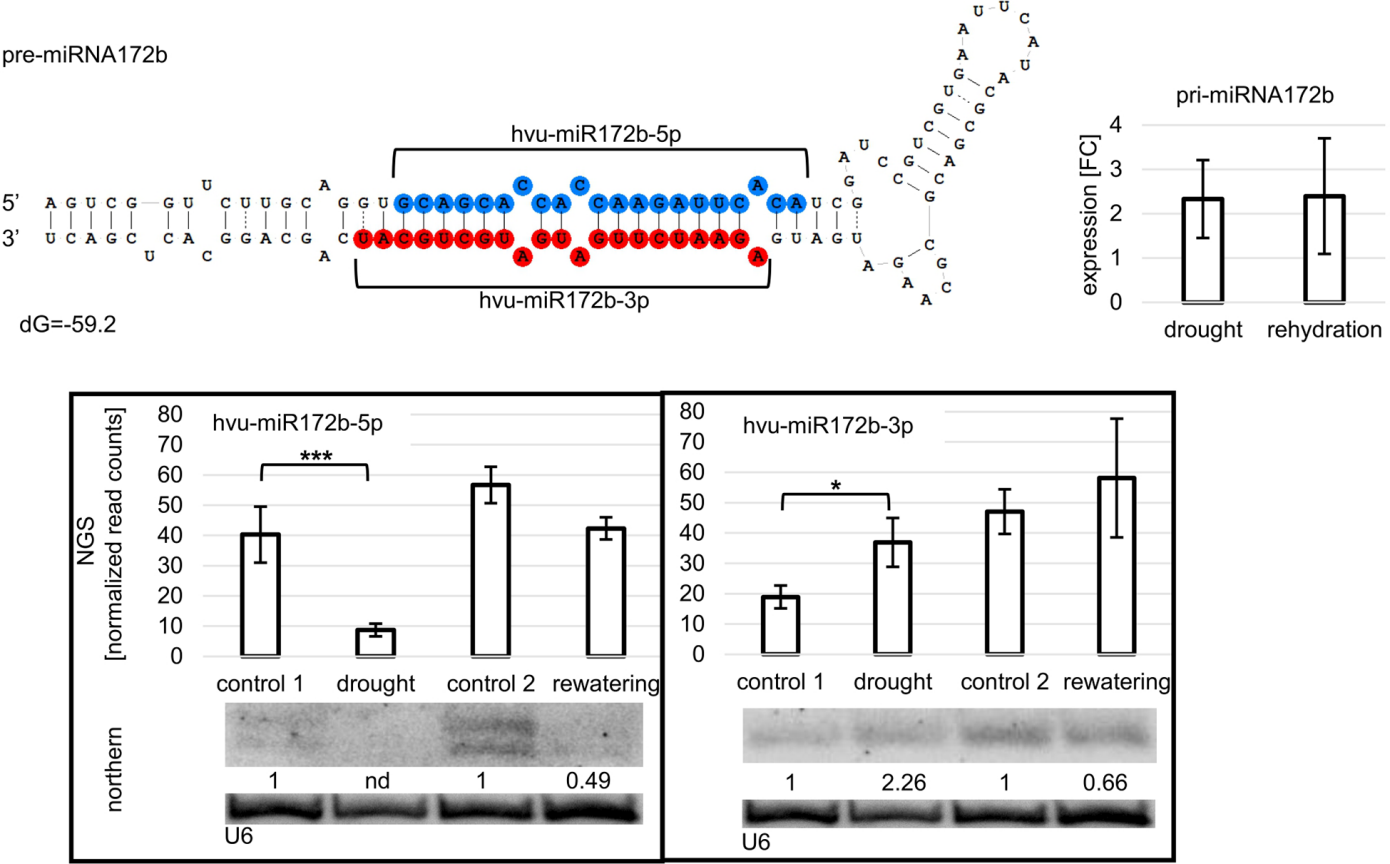
Hvu-miR172b-5p and hvu-miR172b-3p originating from a common precursor are differently expressed during drought. The pri-miRNA172b level is not significantly changed during drought or rehydration. Drought induces the downregulation of miRNA172b-5p, while the same treatment upregulates miRNA172b-3p. After rehydration, the accumulation of both molecules is at the control_2 level. Pri-miRNA expression was revealed by RT-qPCR; the results are shown as FC. Two-tailed Student’s *t*-test was used to calculate the significance of the result. The significance of the observed NGS differences was calculated with the DESeq package in R (***p ≤ 0.001, *p ≤ 0.05). The microRNA hybridization signal was corrected with U6 as a loading control. nd - not detected.

Northern blot hybridization was performed for selected miRNAs to confirm the drought- and/or rehydration-affected level fluctuations revealed by sRNA NGS. Eight miRNAs, ptc-miR6478, osa-miR5072, ata-miR168-3p, bdi-miR166e-3p, ata-miR396c-3p, bdi-miR1432, hvu-miR172b-3p, and hvu-miR172b-5p, differing in their level profile were selected (**Figures 2**, **3**). On each panel, the accumulation revealed by NGS for a given miRNA is displayed on a graph as normalized read counts. The lower northern signal in both stress conditions was observed in the case of three miRNAs: ata-miR168-3p, bdi-miR166e-3p, and ata-miR396c-3p. The Northern blot for one miRNA, ptc-miR6478, revealed a higher drought and rehydration signal. Osa-miR5072 and hvu-miR172b-3p were characterized by an elevated northern signal only in drought. A lower-intensity band only in rehydration was detected for bdi-miR1432. The absence of a northern signal only in drought was detected solely in the case of hvu-miR172b-5p. All the statistically significant accumulation changes induced by drought or rehydration and revealed by NGS are reflected by the expression pattern visible as the northern hybridization signal.

Barley pri-miRNAs accumulation changes during drought and rehydration in the Rolap spring barley genotype were studied before in Swida-Barteczka et al. (2016). The sampled material was the same in both of our studies. This allowed us to compare pri-miRNA data to conserved miRNA accumulation changes revealed in this study. The pri-miRNA–miRNA relationships are presented in **Table 3**. Predominantly, pri-miRNA accumulation changes in either drought or rehydration do not reflect the mature miRNA abundancies in these conditions. Mostly, the changes in pri-miRNA expression are followed by unchanged mature miRNA accumulation.

**Table 3:** Accumulation changes of miRNAs in relation to the cognate pri-miRNAs during drought or rehydration.

pri-miRNA – miRNA relation		drought	rehydration
pri-miRNA = miRNA	pri-miRNA const., miRNA const.	14	20
	pri-miRNA ↓, miRNA ↓	4	2
	pri-miRNA ↑, miRNA ↑	5	_
pri-miRNA < miRNA	pri-miRNA const., miRNA ↑	1	3
	pri-miRNA ↓, miRNA↑	1	0
	pri-miRNA ↓, miRNA const.	20	13
pri-miRNA > miRNA	pri-miRNA ↑, miRNA const.	21	35
	pri-miRNA ↑, miRNA ↓	3	_
	pri-miRNA const., miRNA ↓	6	3

### 3.2 Experimentally validated targets for the drought- and/or rehydration-responsive conserved microRNAs

Degradome libraries from control_1 and drought barley plants were prepared to identify target mRNAs for the conserved barley miRNAs. After library sequencing on the TruSeq system of Illumina, the obtained reads were analyzed to identify target mRNAs. The target mRNAs were identified by miRNA-driven cleavage site scoring. The scoring parameters consisted of degradome raw and normalized reads’ counts ranked as the cleavage sites of a particular cDNA and the cutting potential of the miRNA/candidate target mRNA pair assessed on the miRNA-target alignment quality, where negative points were assigned for sequence mismatches. The compliance score values range from 0 to 18, where the lowest score indicates the best candidate target mRNA (**Supplementary Table 3**; **Supplementary Table 4**). The target plots (t-plots) of each particular cDNA for control (ak) and drought (as) libraries are accessible on the mirEX 2.0 web portal http://www.combio.pl/mirex2/(Degradome data for selected plant species link) (Bielewicz et al., 2012; Zielezinski et al., 2015). The t-plots can be accessed by typing the MLOC number and selecting the library. MLOC sequences are listed in **Supplementary Table 5**. The t-plots can be displayed, depending on user preference, as raw/normalized read counts or average-based values. The t-plots are presented as interactive graphs where each peak is labeled with the cDNA position, the number of counts, and rank in comparison with other identified cleavage sites. The graphs can be enlarged for a better visualization of peaks or downloaded as .txt file or a .png, .jpeg, .pdf, or .svg chart.

**Figure 4:**
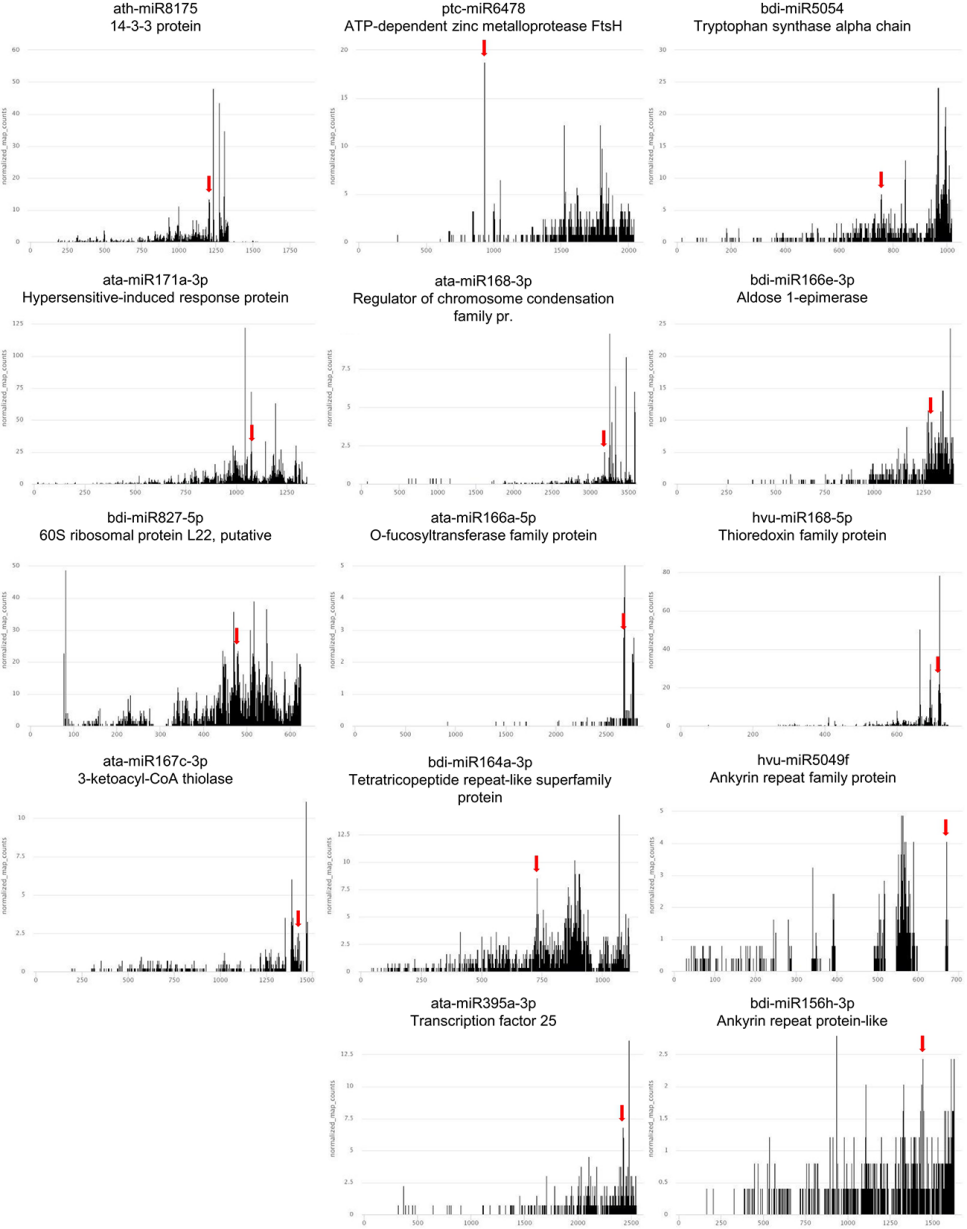
T-plots of the novel mRNA targets whose expression exhibits reversed correlation with cognate miRNA in drought and/or rehydration. The miRNA-driven cleavage site is marked by the red arrow.

**Figure 5:**
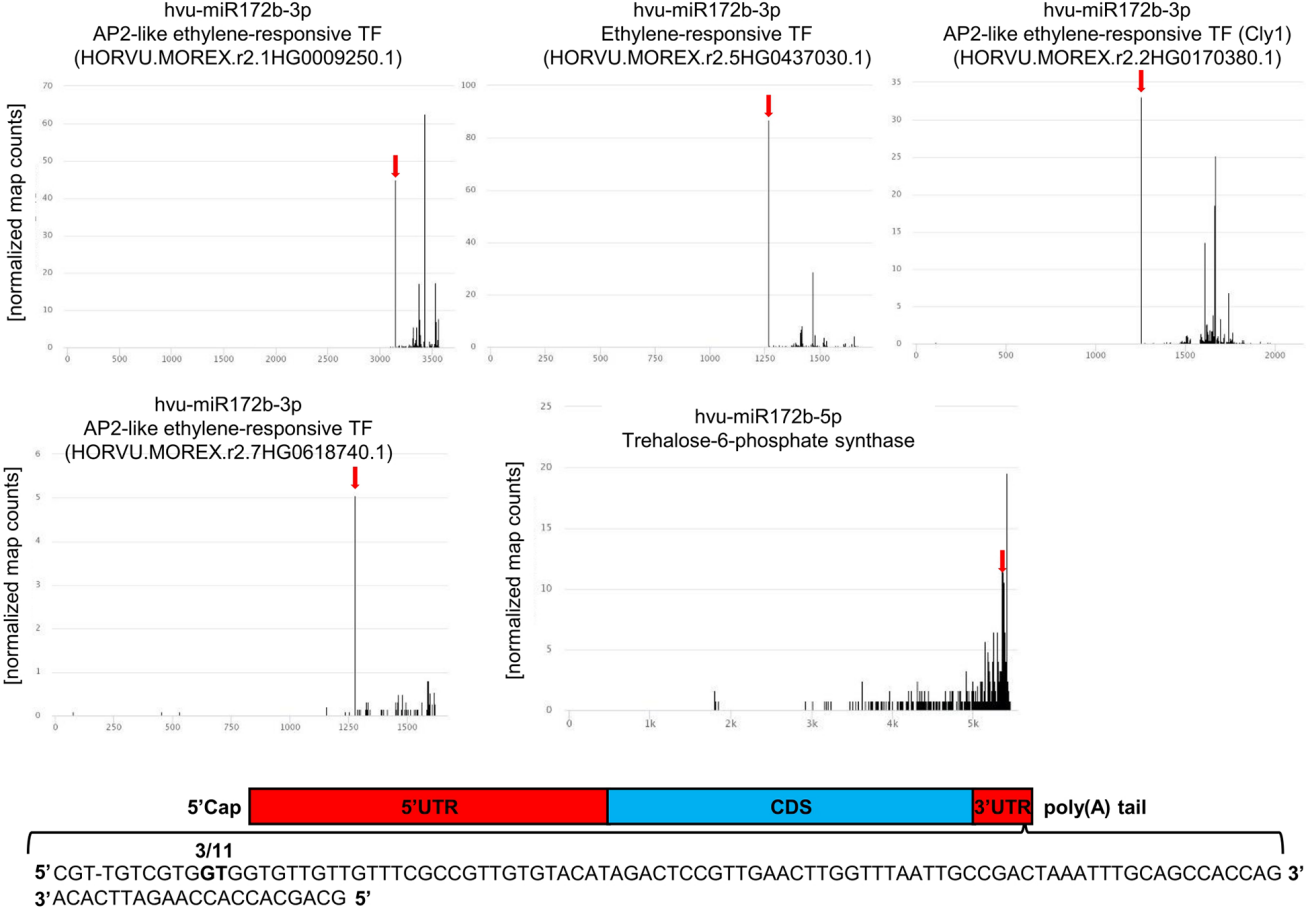
Targets of hvu-miR172b-3p and hvu-miR172b-5p. Four barley AP2-like targets are cleaved by hvu-miR172b-3p. The target of hvu-miR172b-5p is *trehalose-6-phosphate synthase* mRNA. The red arrow indicates the predicted hvu-miR172b-3p or hvu-miR172b-5p cleavage site in four AP2-like or *trehalose-6-phosphate synthase* (*TPS*) mRNAs. The predicted cleavage sites are consistent with the parallel analysis of RNA ends depicted as target plots (t-plots). The names of the microRNA and targeted mRNA are given above each graph; in the case of the AP2-like gene numbers, they are given in brackets. The x-axis on the t-plots represents the nucleotide length of the targeted mRNAs; the y-axis shows the normalized number of reads. In the case of *TPS* mRNA, the cleavage site was validated with the 5’ RACE method; the numbers of clones represent the ratio of positive to all analyzed clones. Two bolded nucleotides within mRNA depict the cleavage site. The sequence represents miRNA:mRNA hybridization and the end of 3’UTR region.

Target mRNAs were identified for 56 out of the 58 miRNAs differentially accumulated during drought or/and rehydration stress. Targets for gma-miR4995 and tae-miR9773 were not identified. For each of the 56 differentially expressed conserved miRNAs, the targets with the lowest compliance score values were selected. The chosen target mRNAs are listed in **Supplementary Table 6**. Usually, one target or two targets with similar compliance score values are presented. The exception is hvu-miR172b-3p where four targets from the same protein family are listed. Altogether, we selected 80 best-quality targets for 56 conserved barley miRNAs differentially accumulated during water shortage stress. There were 41 of the identified targets that were not previously recognized as miRNA-dependent in barley. A functional description of the chosen target mRNA MLOCs is shown in **Supplementary Table 6**, and their gene numbers are listed in **Supplementary Table 7**. T-plots for novel barley targets are presented in **Figures 4**, **5** and **Supplementary Figure 2**.

The expression alterations during the drought and rehydration of the best-quality targets for the 56 differentially accumulated, conserved miRNAs were revealed by RT-qPCR. Target mRNAs whose expression is negatively correlated to the cognate miRNA in drought or rehydration are presented in **Table 4**. There are 15 of these mRNAs that are novel ones (**Figures 4**, **5**). These include targets like *14-3-3 PROTEIN* targeted by ath-miR8175, *ATP-DEPENDENT ZINC METALLOPROTEASE FtsH* targeted by ptc-miR6478, *TRYPTOPHAN SYNTHASE ALPHA CHAIN* targeted by bdi-miR5054, *HYPERSENSITIVE-INDUCED RESPONSE* targeted by ata-miR171a-3p, *O-FUCOSYLTRANSFERASE FAMILY* targeted by ata-miR166a-5p. Despite the low compliance score values of the degradome data or being previously reported as miRNA targets in other studies, the expression pattern of many targets is not negatively correlated to cognate miRNAs. The expression alterations of these targets in drought- and rehydration-treated barley plants are presented in **Supplementary Table 8**. These mRNA targets also include novel targets (**Supplementary Figure 2**).

**Table 4:** Target mRNAs whose expression is controlled by miRNAs in drought and/or rehydration .

microRNA	microRNA expression change [log_2_ FC]		target expression [FC]				target
	drought	rehydration	drought	p-value	rehydration	p-value	
ath-miR8175	2.36	1.89	-1.57	***	1.86	**	14-3-3 protein
ptc-miR6478	1.45	1.12	-4.80	***	-6.43	***	ATP-dependent zinc metalloprotease FtsH
bdi-miR5054	1.03	1.68	-2.03	**	-1.39	**	Tryptophan synthase alpha chain
bna-miR167d	1.37	0.34	-2.56	***	1.75	*	Auxin response factor
hvu-miR172b-3p	1.07	0.26	-2.26	***	-1.03		Ethylene-responsive transcription factor
			-3.08	***	1.03		AP2-like ethylene-responsive transcription factor
			-1.33	***	1.32		AP2-like ethylene-responsive TF (Cly1)
			-1.93	*	-1.11		AP2-like ethylene-responsive transcription factor
hvu-miR156a/b	1.01	0.43	-1.53	*	-1.11	*	Squamosa promoter-binding-like protein
aly-miR164a-5p	0.12	1.88	1.27		-1.61	***	NAC domain-containing protein
bdi-miR156h-3p	1.11	1.48	2.96		-1.28	*	Ankyrin repeat protein-like
ahy-miR159	0.65	0.65	-1.16		-1.09	**	myeloblastosis viral oncogene homolog (MYB) transcription factor
ata-miR171a-3p	-0.93	-1.11	2.30	***	-1.09		Hypersensitive-induced response protein
ata-miR168-3p	-0.96	-1.21	2.99	***	1.75	**	Regulator of chromosome condensation family protein
bdi-miR166e-3p	-1.81	-1.00	1.68	*	1.29	**	Aldose 1-epimerase
bdi-miR827-5p	-1.85	-1.01	3.98	***	1.29		Syg1/Pho81/XPR1-MAJOR FACILITATED SUPERFAMILY1
			-1.16		1.83	***	60S ribosomal protein L22, putative
hvu-miR171-5p	-2.20	-0.69	1.63		1.33		Disease resistance protein (Toll/interleukin-1 receptor (TIR)-nucleotide-binding site (NBS)-leucine-rich repeat (LRR)) family
ata-miR166a-5p	-2.45	-0.93	-1.51	*	1.40	**	O-fucosyltransferase family protein
ata-miR5168-5p	-4.20	-2.07	2.78	***	1.39	*	F-box family protein
cme-miR166g	-0.65	0.06	1.68	*	1.29	**	Aldose 1-epimerase
ata-miR166c-3p	-0.73	0.01	6.70	*	2.68	*	Homeobox leucine-zipper protein
ata-miR5168-3p	-0.82	-0.29	6.70	*	2.68	*	Homeobox leucine-zipper protein
hvu-miR168-5p	-0.87	-0.29	1.29	*	-1.06		Thioredoxin family protein
csi-miR166d	-0.87	-0.24	6.70	*	2.68	*	Homeobox leucine-zipper protein
aqc-miR166c	-0.87	-0.28	6.70	*	2.68	*	Homeobox leucine-zipper protein
ata-miR398f-3p	-1.14	-0.49	1.70	*	1,00		Superoxide dismutase [Cu-Zn]
ata-miR167c-3p	-1.58	0.10	3.73	**	1.33	*	3-ketoacyl-CoA thiolase
osa-miR166e-3p	-1.79	0.20	6.70	*	2.68	*	Homeobox leucine-zipper protein
hvu-miR172b-5p	-2.00	-0.40	3.09	*	2.15	***	Trehalose-6-phosphate synthase
bdi-miR164a-3p	-2.38	-0.47	2.13	*	-1.74	***	Tetratricopeptide repeat-like superfamily protein
hvu-miR5049f	-2.41	-0.53	3.54		-2.56	***	Ankyrin repeat family protein
ata-miR395a-3p	-2.54	0.48	2.38	**	1.45	**	Transcription factor 25
zma-miR168a-3p	0.03	-0.86	2.99	***	1.75	**	Regulator of chromosome condensation family protein
ata-miR156c-3p	-1.25	-1.12	-2.30	***	1.49	**	C2 calcium/lipid-binding and glucosyltransferases, Rab-like GTPase activators and myotubularins (GRAM) domain protein

Target expression was revealed by RT-qPCR; the results are shown as FC. The levels of target mRNAs under the control conditions were assumed to be 1 for upregulated targets or -1 for downregulated targets, and the levels of target mRNAs under stress conditions were quantified in relation to this standard. The significance of the observed changes was tested with a two-tailed Student’s t-test (***p ≤ 0.001, **p ≤ 0.01, and *p ≤ 0.05). Up- and downregulated miRNAs and target mRNAs are marked as red and blue, respectively.

### 3.3 MicroRNA172b-5p and microRNA172b-3p derived from a common precursor are functional molecules targeting genes responsible for flowering timing and drought resistance

Among the drought- and rehydration-deregulated miRNAs are hvu-miR172b-5p and hvu-miR172b-3p. Interestingly, the two miRNAs originate from a common precursor (**Figure 3**), and the mean number of reads from all 12 sRNA libraries is similar and equals 48.88 and 54.07 for hvu-miR172b-5p and hvu-miR172b-3p, respectively. The two miRNAs originating from a common precursor accumulate differentially during drought stress in barley. MiRNA172-5p is downregulated upon drought, and rewatering restores the miRNA172b-5p accumulation level (**Figure 3**). In contrast, miRNA172b-3p level is reversely affected—upregulated in the same stress (**Figure 3**), and rewatering restores the basal accumulation level. The drought-dependent hvu-miR172b-5p and hvu-miR172b-3p accumulation alterations are visible both in NGS results and northern analysis. Interestingly, the pri-miRNA172b level is not statistically increased by drought or rehydration (**Figure 3**) (Swida-Barteczka et al., 2016).

**Figure 6:**
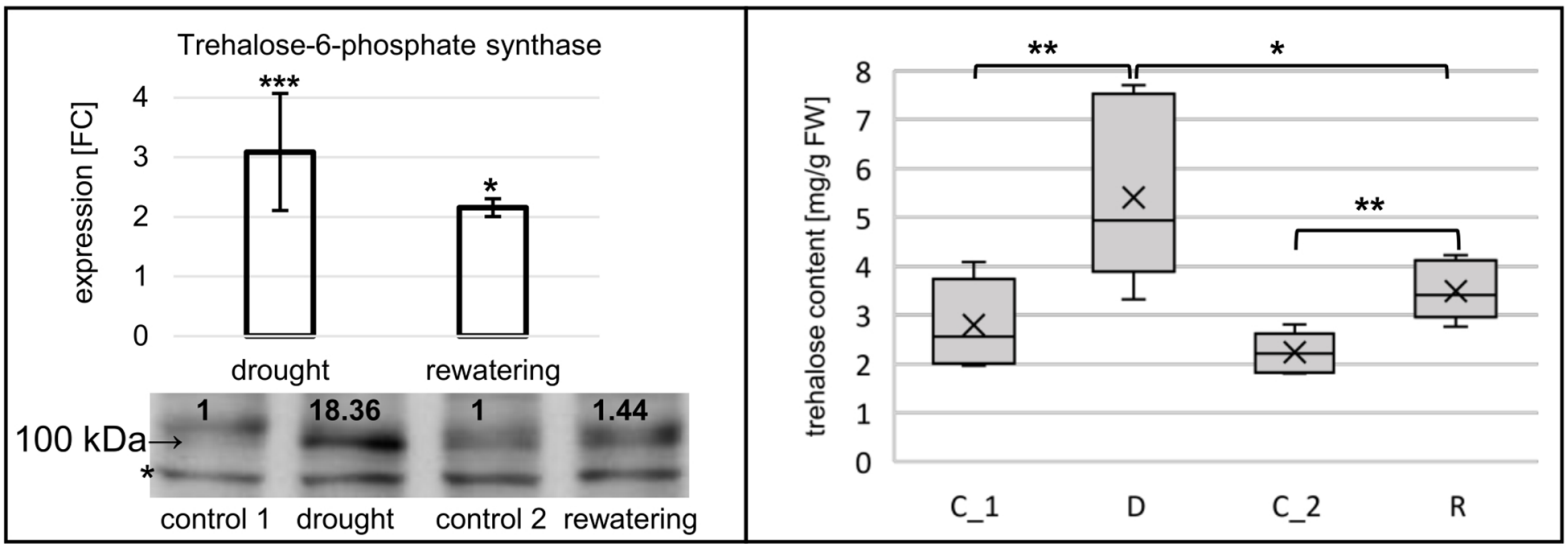
Drought induces trehalose synthesis in barley through the expression regulation of *TPS*. Left panel, the expression of *TPS* is elevated during drought and rehydration at the level of mRNA, while the protein level is strongly increased only during drought. Target mRNA expression was revealed by RT-qPCR; the result is shown as FC. The numbers below the blot image are the relative intensities of the protein band; control signals were treated as 1. The protein signal was corrected with an unspecific signal (*) as a loading control. In the right panel, trehalose content measurements in drought- and rehydration-stressed barley. Trehalose content increases during drought; the change is quickly reversed by rewatering. The significance of the mRNA expression changes and trehalose content differences were calculated with two-tailed Student’s *t*-test (***p ≤ 0.001, **p ≤ 0.01, and *p ≤ 0.05).

For hvu-miR172b-5p and hvu-miR172b-3p, target mRNAs are identified in the degradome data obtained in this study. Hvu-miR172b-3p targets four AP2-like TF genes: AP2-like ethylene-responsive TF (MLOC_77763.3 and HORVU.MOREX.r2.1HG0009250.1), ethylene-responsive TF (MLOC_43575.1 and HORVU.MOREX.r2.5HG0437030.1), AP2-like ethylene-responsive TF (Cly1, MLOC_43830.1, and HORVU.MOREX.r2.2HG0170380.1), and AP2-like ethylene-responsive TF (MLOC_43041.2 and HORVU.MOREX.r2.7HG0618740.1). T-plots for the four AP2-like MLOCs and the number of the identified normalized cleavage site counts (MLOC_77763.3 – ak: 44.95: as: 4.54; MLOC_43575.1 – ak: 86.79, as: 16.1; MLOC_43830.1 – ak: 33.09, as: 24.21; MLOC_43041.2 – ak: 5.05, as: 2.57) differing from the background clearly show the hvu-miR172b-3p-driven cleavage of the four mRNAs (**Figure 5**). The analysis of the degradome data allowed to experimentally identify *TPS* mRNA as a previously-not-known target of hvu-miR172b-5p. The predicted cleavage site is consistent with the *TPS* t-plot (**Figure 5**). The 5’ RACE experiment also confirmed the hvu-miR172b-5p-targeted sites (**Figure 5**). Therefore, we conclude that the hvu-miR172b-5p is a functional molecule. The expression levels of hvu-miR172b-3p and hvu-miR172b-5p induced by drought and rehydration reversely correlate with their target expressions. The upregulated level of hvu-miR172b-3p correlates with the lowered expression of all four targeted AP2-like mRNAs during drought (**Table 4**). The expression of hvu-miR172b-3p and four targeted AP2-like mRNAs returns within 6 h after rehydration to control values. In contrast, drought downregulates the level of hvu-miR172b-5p that is followed by higher levels of *TPS* mRNA and protein (**Table 4**; **Figure 6**, left panel). Interestingly, the level of *TPS* mRNA is still above control levels 6 h after rehydration, while the protein level already dropped. *TPS* is an enzyme converting uridine diphosphate glucose (UDP-glucose) and glucose-6-phosphate to trehalose-6-phosphate in the biosynthesis of trehalose. The measurement of trehalose content in drought and rehydration-treated barley revealed its increase in drought-treated plants, followed by a rapid decrease after rewatering, which is in accordance with trehalose-6-phosphate synthase protein–level fluctuations (**Figure 6**).

### 3.4 Flowering is accelerated in drought-treated plants

**Figure 7:**
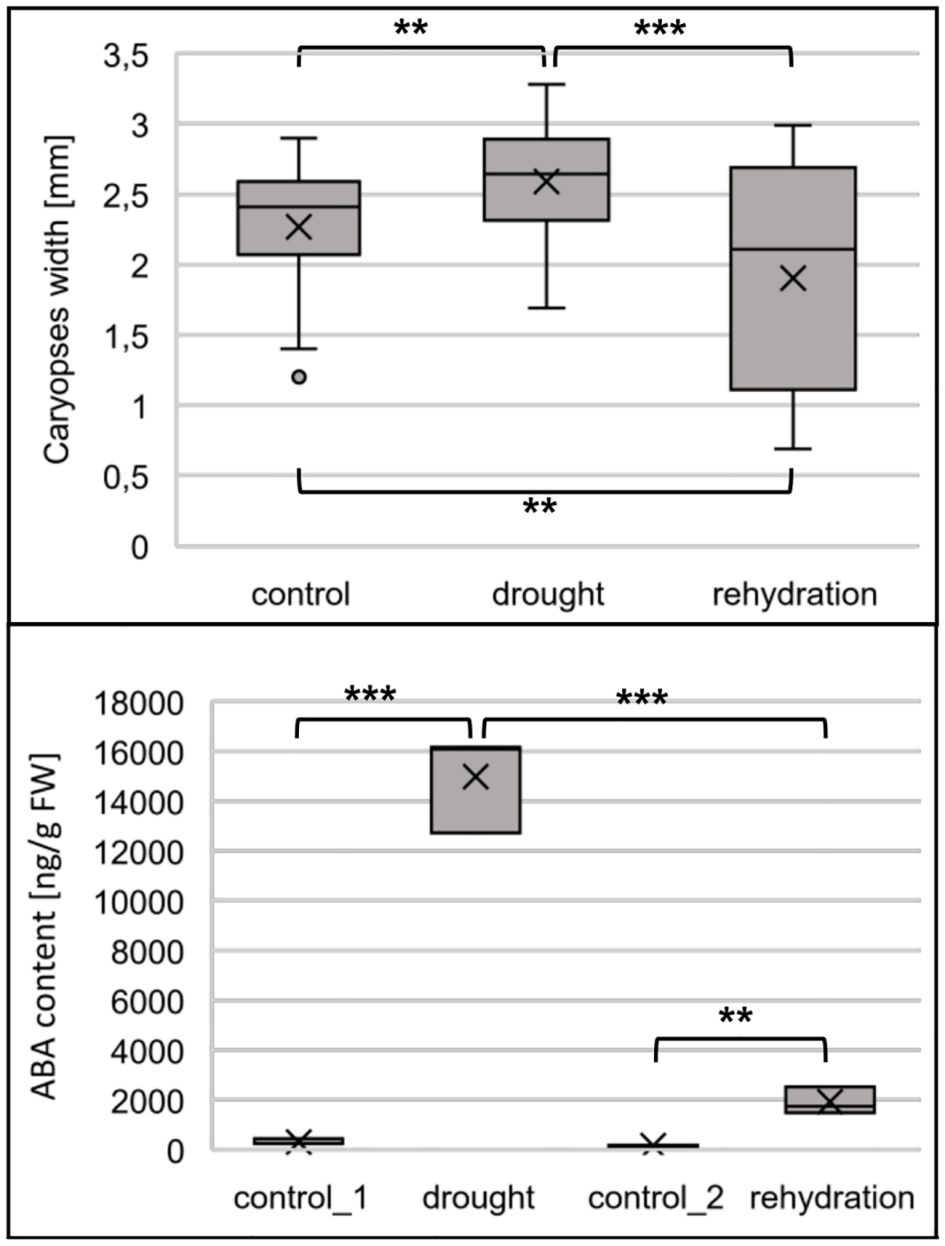
Drought accelerates flowering in barley. Upper panel, caryopses developing in drought- stressed barley are larger than control ones. Caryopses isolated from rehydration-treated plants are smaller. Lower panel, barley reacts to drought with elevated ABA biosynthesis; the biosynthesis is decreased after rehydration. The significance of the presented differences were tested with two-tailed Student’s *t*-test (***p ≤ 0.001, **p ≤ 0.01).

The upregulation of miRNA172-3p and the resulting decrease of AP-2 like gene expression are the determinants of the plant entering into a generative phase of growth. In addition, the intermediate of trehalose synthesis, trehalose-6-phosphate, a direct biosynthetic of TPS, accelerates flowering. Therefore, we tested whether drought applied to barley at the flag leaf appearance, stage 39–41 of the Zadoks cereal development decimal code, accelerates flowering. The accelerated flowering was stated as larger caryopses developed from fertilized flowers. Caryopses isolated from drought-stressed flowers were significantly larger than caryopses obtained from well-watered plants (**Figure 7**, upper panel). Interestingly, caryopses obtained from rehydration-treated plants were generally smaller than those isolated from control or drought-treated plants. Thus, we conclude that flowering and fertilization in drought-treated barley occur earlier than in optimally watered plants. Moreover, rehydration ceases processes leading to flowering acceleration. As drought stress is characterized by increased ABA accumulation, we measured its content in studied plants. In control_1 and control_2 samples, the ABA content was 304 and 158 ng/g FW, the drought stress increased the ABA content to 14991 ng/g FW, and the ABA content dropped to 1913 ng/g FW within 6 h after rewatering (**Figure 7**, lower panel).

## 4 Discussion

### 4.1 A small fraction of conserved microRNAs is drought- and rehydration-responsive

In this study, we identified conserved miRNAs in barley plants, genotype Rolap treated with severe drought and after 6 h of rehydration. Altogether, we identified 150 conserved barley miRNAs, and 49 of them were differentially accumulated during drought. From this, we conclude that the accumulation of most of the conserved barley miRNAs is stable during drought. The general tendency of the differentially accumulated 49 miRNAs is toward the decrease of their abundancy: 38 miRNAs were downregulated, and only 11 were upregulated. Similar observations were reported for other barley varieties in drought experiments (Hackenberg et al., 2015; Fard et al., 2017; Ferdous et al., 2017). Despite the fact that the experiments performed by others were conducted on diverse spring barley cultivars and the growth stadium or drought stress duration was different, the general decreasing tendency of miRNA levels is the same. Importantly, some conserved barley miRNAs can be highlighted as showing a common accumulation pattern during drought independently on the cultivar, growth stadium, or drought duration. The commonly upregulated miRNAs in this and other studies are two miRNAs: gma-miR6300 and ppt-miR894, and the downregulated miRNAs are bdi-miR166e-3p and ata-miR1432-5p. In all studies, the miRNA with consistently unchanged expression is bdi-miR393a (Hackenberg et al., 2015; Ferdous et al., 2017). However, the general competence of barley plant to regulate individual miRNA expression as a reaction to drought in most cases depends on the cultivar or developmental stage of the plant. An example is hvu-miR156a/b, which is upregulated in Rolap and in drought-tolerant Yousef but not in drought-susceptible Morocco 9-75, while hvu-miR168-5p and hvu-miR159a/b are downregulated in Rolap and Morocco 9-75 but not in Yousef (Fard et al., 2017). Cultivar dependent-induced accumulation can also be observed in the case of aly-miR164a-5p, whose expression is not induced in the case of Rolap, Golden Promise, and Morocco 9-75, while it is upregulated in Yousef during drought and in Rolap after rehydration (Hackenberg et al., 2015; Fard et al., 2017). We also found a set of miRNAs that were reported as drought-responsive in other cultivars but are not responsive to drought in Rolap. These miRNAs are sbi-miR156e, ata-miR396a-5p, bdi-miR444b, aly-miR167a-5p, ahy-miR167-5p, hvu-miR444b, gma-miR393h, rgl-miR5139, hbr-miR166a, hvu-miR5048a/b, hvu-miR166a/b/c, and aly-miR160a-5p (see **Supplementary Table 2**) (Hackenberg et al., 2015; Fard et al., 2017; Ferdous et al., 2017). The mentioned miRNAs were up- or downregulated, and, in some cases, their expression is reported to be unchanged in one but not all of the cited works. In these cases, it is difficult to conclude if the inconsistencies depend on differences between cultivars or the developmental stage of the tested barley plants.

Our study was predominantly focused on a wide analysis of conserved barley miRNAs, and altogether, we identified new, till-now non-reported 50 drought- and/or rehydration-responsive miRNAs and 61 as non-responsive (see **Table 2**; **Supplementary Table 2**). We were also interested if the severe drought-induced changes in conserved miRNA accumulation are reversible after irrigation. In the case of Rolap, the accumulation of most of the drought-responsive miRNAs turned out to be sensitive to watering conditions. This observation can be concluded from the fact that, in our study, the expression of the 33 out of the 49 differentially accumulated miRNAs during drought was reversed to control levels within 6 h after rehydration. Moreover, rehydration can alter the accumulation of some drought-insensitive miRNAs, which is observed in the case of four upregulated and five downregulated miRNAs (see **Table 2**; **Supplementary Table 2**).

Barley pri-miRNA expression patterns during drought and rehydration were studied before in the two genotypes Rolap and Sebastian (Swida-Barteczka et al., 2016). The comparison of individual pri-miRNA expression fluctuations with data presented in this study revealed that, in many cases, pri-miRNA accumulation does not reflect the mature miRNA accumulation changes in these growth conditions. This is in agreement with Arabidopsis pri-miRNA and mature miRNA accumulation study testing many abiotic stresses (Barciszewska-Pacak et al., 2015). The discrepancies can be explained by complex pri-miRNA processing, which includes splicing, alternative splicing, alternative polyadenylation, pre-miRNA processing, or degradation as well as mature miRNA stability or export out of the cell or tissue (Kruszka et al., 2014; Bajczyk et al., 2023).

### 4.2 A set of target mRNAs is regulated predominantly by microRNAs during drought and rehydration

We predicted and experimentally validated target mRNAs for all but two conserved barley miRNAs identified in the Rolap variety. A total of 58 miRNAs showed altered expression during drought and/or rehydration, and, for the 56 of them, we found one or more good-quality target mRNAs using computational and experimental PARE analysis. The influence of drought or rehydration on the accumulation of these target mRNAs was confirmed by RT-qPCR. Some target mRNAs are recognized by more than one drought-responsive miRNA from the same family. Altogether, the expression levels of 63 target mRNAs were tested. The expression levels of 31 of them are reversely correlated with their cognate miRNA accumulation during drought or rehydration stress. Additionally, the negative correlation between the accumulation levels of miRNAs and their targets induced by drought decreases after rehydration in the case of 28 out of the 31 reversely correlated target mRNAs. Therefore, we conclude that changes in the expression of at least 28 target mRNAs are controlled mainly by the assigned miRNAs.

This study presents a wide analysis of target mRNAs for drought-responsive, conserved miRNAs. There are 46 of the identified target mRNAs that are novel for barley. Till now, such detailed analysis of target mRNAs and their cognate drought-responsive miRNAs was reported for six novel barley miRNAs (Smoczynska et al., 2020). Among the target mRNAs whose expression in barley is regulated predominantly by drought-responsive miRNAs are 15 novel targets. The novel barley target of drought-responsive miRNA is *14-3-3 PROTEIN* mRNA targeted by ath-miR8175. The 14-3-3 protein overexpression in Arabidopsis results in cold tolerance and the overexpression of many cold or oxidative stress-response proteins (Visconti et al., 2019). Another novel target is *ATP-DEPENDENT ZINC METALLOPROTEASE FtsH* mRNA targeted by ptc-miR6478. The gene encodes thylakoid membrane protease involved in the biogenesis of thylakoid membranes and the degradation and assembly of protein complexes in the photosynthetic electron-transport pathways (Kato and Sakamoto, 2018). Another chloroplastic novel target is *TRYPTOPHAN SYNTHASE ALPHA CHAIN* mRNA cleaved with the help of bdi-miR5054. Plastids are the main compartment of tryptophan biosynthesis in plants (Kriechbaumer et al., 2008). This may be relevant to the fact that, in *Lupinus termis*, external Trp application also alleviates the growth suppression induced by drought by the enhancement of photosynthetic pigment activity (Sadak and Ramadan, 2021). *HYPERSENSITIVE-INDUCED RESPONSE* novel target mRNA guided by ata-miR171a-3p encodes a plasma membrane–localized protein whose role is recognized in the development of lesions in leaves of rice, barley, or Arabidopsis in response to biotic stress (Rostoks et al., 2003; Zhou et al., 2010). The target mRNA important for pollination is *O-FUCOSYLTRANSFERASE FAMILY* cleaved with the assistance of ata-miR166a-5p, which is necessary for effective pollen tube penetration through the stigma [Smith et al., 2018]. Among the previously reported targets of drought-responsive miRNAs are two *CALMODULINS* mRNAs targeted by two miRNAs: ata-miR1432-5p and bdi-miR1432. CALMODULINS bind calcium ions and are messengers in signaling networks. In barley, they are responsive to salinity, high potassium, or osmotic stresses [Cai et al., 2022]. Interestingly, the third target of these miRNAs is novel, and it is *2-OXOGLUTARATE (2OG) AND FE(II)-DEPENDENT OXYGENASE SUPERFAMILY PROTEIN* (2-OGO) mRNA. 2-OGO is a plant immunity suppressor in Arabidopsis and barley and defines susceptibility to *Fusarium graminearum* (Low et al., 2020). Another novel target that is involved in plant resistance to biotic stresses is *CALRETICULIN* mRNA targeted by aly-miR166a-5p. CALRETICULIN is calcium ion–binding protein residing in endoplasmic reticulum. Its biological role is to regulate plant immunity through salicylic acid–mediated defense responses (Qiu et al., 2012). The novel targets identified by us are connected to plant stress tolerance or chloroplast integrity and function.

### 4.3 MiRNA172b-3p directs cleavage of barley AP2-like mRNAs

One of the drought-upregulated miRNAs is miRNA172b-3p. Its accumulation drops down to the control level within 6 h after rehydration. The miRNA 172 members target the mRNAs of AP2-like TF family. In barley, genotype Rolap, we identified four members of AP2-like TFs as the highest-scored and experimentally validated targets of miRNA172b-3p (see **Figure 5**; **Supplementary Table 6**). Drought elevates the accumulation of miRNA172b-3p, while all AP2-like gene expressions are downregulated at the level of mRNA. Moreover, rehydration restores the expression of all tested AP2-like TFs and their regulatory miRNA to basal levels (see **Table 4**). Arabidopsis, maize, or rice AP2-like targets of miRNA172 are reported to be downregulated by translational inhibition (Aukerman and Sakai, 2003; Chen, 2003; Chuck et al., 2007). However, in the case of rice, there is a report showing that the AP2-like target of miRNA172, SUPERNUMERARY BRACT (SNB), is directed to cleavage (Zhu et al., 2009). In the case of barley Cly1 (one of the barley AP2-like TF family members), miRNA172-driven cleavage was reported. However, in another study, the miR172-directed cleavage products of Cly1 transcripts were reported to be rare and their presence was assumed as not being indicative of miR172-directed mRNA cleavage. It was suggested that translational inhibition is a predominant form of Cly1 downregulation (Nair et al., 2010; Anwar et al., 2018). In the case of *H. vulgare*, genotype Rolap, we report a high number of reads originating from the cleavage of *Cly1* mRNA and other AP2-like family members. Therefore, we are leaning toward the miRNA-directed cleavage of AP2-like targets in barley.

### 4.4 Flowering acceleration by miRNA172b-3p/AP2-like module

AP2-like roles are widely recognized in setting floral patterning (Aukerman and Sakai, 2003; Chen, 2003; Chuck et al., 2007; Brown and Bregitzer, 2011). The miRNA172-AP2-like module also regulates flowering timing. The maize AP2-like target of miRNA172, *GLOSSY15* (*GL15*), is responsible for the maintenance of the juvenile phase visible as an elevated number of juvenile leaves and the delay of reproductive development in *gl15* (Lauter et al., 2005). The maize homolog of TOE1 - *ZmRap2.7*, a target of miRNA172, also has strong influence on flowering timing; its overexpression results in delayed flowering, while knockdown leads to early flowering (Salvi et al., 2007; Li et al., 2019). As mentioned earlier in the case of barley, genotype Rolap, we observed that the drought- induced upregulation of miRNA172b-3p accumulation is followed by the decrease of all four identified AP2-like target mRNAs. The negative correlation is ceased 6 h after the rehydration of plants, where the expression of miRNA172b-3p and four AP2-like targets are at control levels. From these results, we expected that flowering in drought-stressed barley should be accelerated when compared to well-watered plants. Caryopses’ size at the early stages of their development measured in drought-stressed plants is larger when compared to well-watered plants (see **Figure 7**, upper panel). This indicates that pollination occurred earlier in drought. Interestingly, the rapid changes in miRNA172b-3p/AP2-like module expression caused by drought followed by rehydration resulted in generally smaller caryopses than in drought-treated and well-watered plants. This can be explained by the fact that, after rehydration, the flowering process slowed down and these consecutive perturbations resulted in smaller caryopsis size. These data indicate that the adaptation of the flowering induction pathway to the watering regimes is limited. Flowering acceleration in response to drought is known as one of the plant strategies helping to avoid the detrimental effects of this stress (Kazan and Lyons, 2016). Interestingly, plant response to drought regarding flowering timing varies within specimen as was shown for Arabidopsis grown in different photoperiods (Riboni et al., 2013). Various Arabidopsis ecotypes also react to drought in various manners, postponing or accelerating flowering (Kenney et al., 2014; Schmalenbach et al., 2014). Continuous, mild drought in barley has been reported to delay heading and flowering and resulted in a decreased number of spikes and seeds (Gol et al., 2021). However, severe drought along with the lower biomass accumulation accelerates the caryopsis maturation and increases the content of protein in mature caryopses (Li et al., 2020). Increased ABA biosynthesis is one of the main plant responses to drought stress (Soma et al., 2021). In drought-treated barley, genotype Rolap, the ABA content increased 49 times, while, in rehydrated plants, the ABA dropped but was still 12 times increased (see **Figure 7**.). Higher ABA content in adult barley plants (cultivar Sebastian) increased drought tolerance as was shown for *ABA INSENSITIVE 5* mutant *havbi5.d* (Collin et al., 2020). The *havbi5.d* is characterized by faster stomata closure and increased water content after drought treatment. The phenotype results from the amplification of the ABA signaling pathway and the activation of ABA-related genes. Whether the accelerated flowering induced by hvu-miR172b-3p/172b-5p-dependent regulatory module is the ABA-dependent process is not known and awaits further studies.

### 4.5 Trehalose biosynthesis is controlled by the miRNA172b-5p/TPS regulatory module

MiRNA172b-3p (regarded as the main miRNA) and miRNA172b-5p (regarded as miRNA*) originate from a common pri-miRNA172b precursor. The read GCAGCACCACCAAGATTCACA (miRNA172b-5p) and read AGAATCTTGATGATGCTGCAT (miRNA172b-3p) align to the barley genome (Hordeum_vulgare.MorexV3_pseudomolecules_assembly) in a single locus. In control conditions, both miRNAs exhibit a similar expression level as concluded from sRNA NGS data and northern hybridization (see **Figure 3**). However, in drought conditions, the miRNA172b-3p level is upregulated while miRNA172b-5p decreases. The level of pri-miRNA172b remains unchanged in control and drought conditions. Rehydration restores the miRNA172b-5p/3p expression to control levels. This suggests that yet-unknown posttranscriptional processes affect the final level of both miRNA species derived from the same precursor in response to environmental cues. miRNA* accumulation was observed in the case of barley, rice, or orchid (Peng et al., 2013; Aceto et al., 2014; Grabowska et al., 2020). Noteworthy, it was reported that the proportion between the accumulation of miRNA or its cognate miRNA* varies between specific tissues or the developmental stages of a specific organ. The mechanism discriminating between miRNA or miRNA* accumulation involves differences between intermolecular thermostabilities at the 5’ end of duplex strands. Strand selection depends on HYL1, and the strand with lower 5’ end thermostability is preferentially retained (Eamens et al., 2009). It is not known to what extent abiotic stresses like drought, salinity, and heat stress affect the thermostability of miRNA/miRNA* duplexes. However, this can be one of the possible mechanisms regulating the level of miRNA172b-5p and miRNA172b-3p during drought and rehydration.

We identified barley *TPS* mRNA as a target of miRNA172b-5p. The decrease of miRNA172b-5p accumulation induced by drought is correlated with *TPS* upregulation that is visible at the level of mRNA and particularly strongly at the level of protein. Rehydration lowers the *TPS* expression that is observed as a rapid decrease of protein accumulation. The computational analysis and experimental validation of degradome data as well as 5’RACE experiments point to the miRNA172b-5p-driven cleavage of barley *TPS* mRNA. Thus, we discovered a novel miRNA/mRNA module regulating trehalose synthesis in response to drought.

TPS is one of the key enzymes in trehalose biosynthesis. In barley, the trehalose content mirrors TPS abundance: it is accumulated in drought-stressed plants and significantly drops down 6 h after rehydration. Trehalose is a key organic osmolyte effectively involved in plant abiotic stress tolerance. It is believed to play a protective role against plant desiccation. It also regulates stomata aperture. Transgenic plants overexpressing the genes of microbial trehalose biosynthesis display enhanced tolerance to drought (Kosar et al., 2019). The trehalose biosynthetic pathway also has an established role in plant signaling, especially important during generative development. Interestingly, the Arabidopsis knockdown mutant of *TPS1* flowers extremely late (Wahl et al, 2013). This is in agreement with our data showing that the upregulation of barley *TPS* and the downregulation of AP2-like TF family members lead to the acceleration of flower development. Improving the trehalose biosynthesis by the overexpression of the final enzyme of this pathway in maize also has positive results visible as substantially higher kernel field production, especially visible during drought (Nuccio et al., 2015). In this attempt, the rice *TPP* was expressed in maize under a floral-specific promoter. In our experiments, enhanced trehalose biosynthesis results in larger caryopses at the early stage of development. It would be interesting to test the effect of trehalose on kernel field production in barley. Trehalose conversion to glucose also improves drought tolerance as was shown in Arabidopsis overexpressing *AtTRE1* (Van Houtte et al., 2013). Such plants have better water-retaining capacity resulting from increased sensitivity toward ABA-dependent stomatal closure. Reversed effects are visible in *attre1* Arabidopsis mutants. It would be interesting to test whether barley *TRE1* overexpression has positive effects on productivity and drought tolerance.

**Figure 8:**
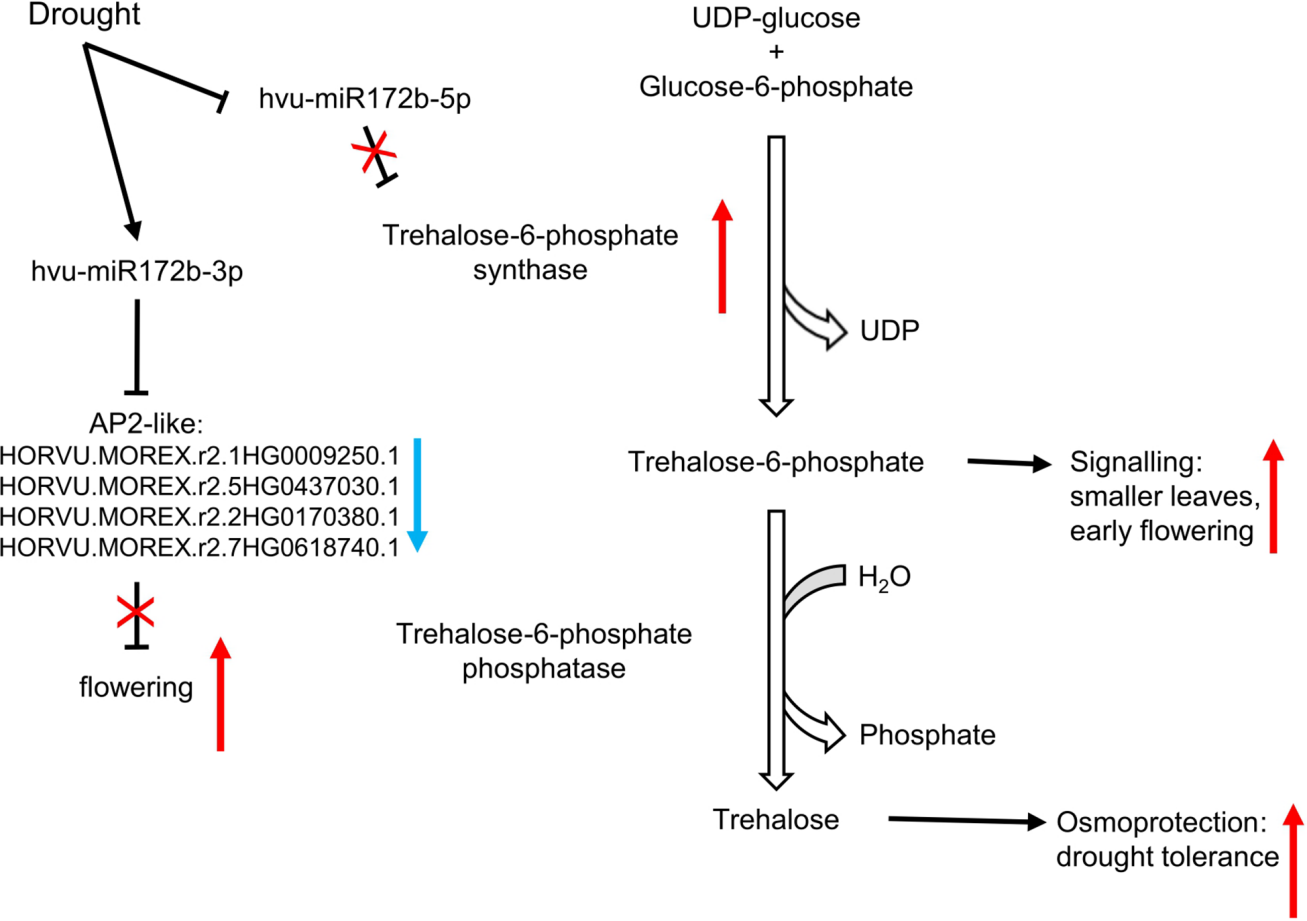
Drought as a stimulus of induced flowering *via* hvu-miR172b-5p/trehalose-6-phosphate synthase and hvu-miR172b-3p/AP2-like modules. The two microRNAs act consistently to speed up flowering by distinct pathways.

In our work, we propose a model where two miRNAs originating from a common precursor, miRNA172b-3p and miRNA172b-5p, act together to accelerate flowering and increase the trehalose level after drought stress in barley. The level of both miRNAs results in controlling AP2-like TFs and the key enzyme of trehalose biosynthesis TPS (**Figure 8**). The hvu-miR172b-3p and its miRNA*-hvu-miR172b-5p, *via* their targets, induce earlier flowering and increased osmoprotective trehalose synthesis as a response to rapidly emerged drought stress after a long period of optimal development. Our study is the first report showing the importance of drought- and rehydration-regulated miRNAs in trehalose biosynthesis. The regulation of trehalose content by miRNAs during drought might be a new perspective in obtaining drought-tolerant barley and other plants like rice, rye, wheat, or maize. Nevertheless, further studies are required to test this strategy. Our repository also provides other interesting miRNA and target mRNA modules that are drought- and rewatering-responsive whose importance for barley fitness was not studied before.

## Data availability statement

The datasets presented in this study can be found in online repositories. The names of the repository/repositories and accession number(s) can be found in the article/**Supplementary Material**.

## Author contributions

Conceptualization, ZS-K, AS-B, and AJ; Methodology, AS-B, KK, AP, and PN; Formal analysis, AS-B and WK; Investigation, AS-B; Writing, original draft preparation, AS-B; Writing, review and editing, AS-B, ZS-K, and AJ; Visualization, AS-B and WK; Supervision, AS-B, ZS-K, and AJ; Project administration, ZS-K and KK; Funding acquisition, ZS-K and KK; Software, WK. All authors contributed to the article and approved the submitted version.
