# Supplementary material for: MicroRNA172b-5p/trehalose-6-phosphate synthase module stimulates trehalose synthesis and microRNA172b-3p/AP2-like module accelerates flowering in barley upon drought stress
Source: Front Plant Sci. 2023 Mar 6;14:1124785. doi: 10.3389/fpls.2023.1124785 (PMC10025483; doi:10.3389/fpls.2023.1124785)
Supplement: Supplementary file 1 [file Table_1.docx]

**Supplementary Table 1.** List of probe and primer sequences used in the study.

| **name** | **sequence** | **usage** |
| --- | --- | --- |
| OS159 | ATGCAGCATCATCAAGATTCT | probe hvu-miR172b-3p |
| OS280 | ACCAACTGAGCTAAGGTCGG | probe ptc-miR6478 |
| OS281 | TGTGAATCTTGGTGGTGCTGC | probe hvu-miR172b-5p |
| OS287 | CTTCCCACAGCTTTCTTGACC | probe ata-miR396c-3p |
| OS297 | GGGAATGAAGCCTGGTCCGAG | probe bdi-miR166e-3p |
| OS298 | ATTCACTTGGTGCAAGGCGGG | probe ata-miR168-3p |
| OS325 | TGGCGACTCCGCTGGGGA | probe osa-miR5072 |
| OS332 | GTCGGTGTCATCTCTCCTGAA | probe bdi-miR1432 |
| OS548 | GGACCCCAAACAGAAGGAGG | RT-qPCR F MLOC_40499.1 target ptc-mir6478 |
| OS549 | AGTCGAGGAAGTTGGCCATG | RT-qPCR R MLOC_40499.1 target ptc-mir6478 |
| OS550 | TGCCGTGCGTGTTTTCTTTT | RT-qPCR F MLOC_54932.2 target ptc-mir6478 |
| OS551 | CCGATCAGCCGTACGTACAC | RT-qPCR R MLOC_54932.2 target ptc-mir6478 |
| OS552 | TGAGGATGTGGTGTGGTGTG | RT-qPCR F MLOC_77986.2 target gma-mir5368 |
| OS553 | AGCCTGGTAAGTGCTCATACAG | RT-qPCR R MLOC_77986.2 target gma-mir5368 |
| OS554 | TCTCTGGGACGCCATGTAC | RT-qPCR F MLOC_67486.1 target osa-mir5072 |
| OS555 | CCGACGTTGTTCATCTCCCA | RT-qPCR R MLOC_67486.1 target osa-mir5072 |
| OS558 | AACCCATGCCACTCTTCTCG | RT-qPCR F MLOC_77763.3 target hvu-miR172b-3p |
| OS559 | GGTGTTGGTTGCTTGACGAC | RT-qPCR R MLOC_77763.3 target hvu-miR172b-3p |
| OS562 | ACCGTAGCATGCTTTCTGGT | RT-qPCR F MLOC_63404.1 target ata-miR168-3p, zma-miR168a-3p |
| OS563 | TCTAACTCACTGACCACGCG | RT-qPCR R MLOC_63404.1 target ata-miR168-3p, zma-miR168a-3p mir168-3p |
| OS564 | TTCCCCAATGCCATCAACCA | RT-qPCR F MLOC_74153.1 target bdi-mir166e-3p, cme-miR166g |
| OS565 | TGACTCCTTTCTTCTGGTCGT | RT-qPCR R MLOC_74153.1 target bdi-mir166e-3p, cme-miR166g |
| OS570 | GCGGAAGAGACGTTGACAGA | RT-qPCR F MLOC_61603.1 target ata-miR166c-3p, ata-miR5168-3p, csi-miR166d, aqc-miR166c, osa-miR166e-3p |
| OS571 | GATTCACCAGACCACAGGCA | RT-qPCR R MLOC_61603.1 target ata-miR166c-3p, ata-miR5168-3p, csi-miR166d, aqc-miR166c, osa-miR166e-3p |
| OS572 | AGTTTCTGTCCAAGGCCACC | RT-qPCR F MLOC_79063.3 target ata-miR166c-3p, ata-miR5168-3p, csi-miR166d, aqc-miR166c, osa-miR166e-3p |
| OS573 | GAGGATGTCGGCAACTTTGG | RT-qPCR R MLOC_79063.3 target ata-miR166c-3p, ata-miR5168-3p, csi-miR166d, aqc-miR166c, osa-miR166e-3p |
| OS574 | GTCATCTGTCACCTTGGGCA | RT-qPCR F MLOC_43714.2 target hvu-miR168-5p |
| OS575 | GGTAACATCAGCCAAACCAAAA | RT-qPCR R MLOC_43714.2 target hvu-miR168-5p |
| OS576 | TTGCCAACCCAGACTTCCAG | RT-qPCR F MLOC_67890.1 target ata-mir166d-5p, aly-miR166a-5p |
| OS577 | CCCAGGTCTCTTCGGCAAAT | RT-qPCR R MLOC_67890.1 target ata-mir166d-5p, aly-miR166a-5p |
| OS580 | GAAAGCCGGCAAAGAAGACC | RT-qPCR F MLOC_11773.1 target hvu-miR172b-5p |
| OS581 | AACGGCGAAACAACAACACC | RT-qPCR R MLOC_11773.1 target hvu-miR172b-5p |
| OS584 | CTCTGGGGTTTCTGGCGTAG | RT-qPCR F MLOC_18231.1 target ata-miR156c-3p |
| OS585 | GGCCAGGGACATGATCAACA | RT-qPCR R MLOC_18231.1 target ata-miR156c-3p |
| OS586 | GGGCTCTGCTCAAGGTGG | RT-qPCR F MLOC_69288.1 target bdi-miR408-3p |
| OS587 | CACAAGTGCGCGTCAAAGAA | RT-qPCR R MLOC_69288.1 target bdi-miR408-3p |
| OS596 | TGGTCAATGCGTCGCGGGAGC | RT-qPCR F MLOC_61497.1 target ata-mir1432-5p, bdi-miR1432 |
| OS597 | CTGCGGTACACGGGCTCGTC | RT-qPCR R MLOC_61497.1 target ata-mir1432-5p, bdi-miR1432 |
| OS604 | GCCCCGGATCTTCAGCAT | RT-qPCR F MLOC_11199.6 target gma-miR156k, cme-miR156j |
| OS605 | GTGATCATCTAGGGGTGGCC | RT-qPCR R MLOC_11199.6 target gma-miR156k, cme-miR156j |
| OS606 | GTCACTGGCTGGTTCCTCTC | RT-qPCR F MLOC_43575.1 target hvu-miR172b-3p |
| OS607 | AGTAATGGACAACCGCATGA | RT-qPCR R MLOC_43575.1 target hvu-miR172b-3p |
| OS608 | GAGAGTGACCAGCAAGACAGAA | RT-qPCR F MLOC_20244.2 target ata-mir168-3p, zma-miR168a-3p |
| OS609 | CAACCTTGAACTGGCAGCAT | RT-qPCR R MLOC_20244.2 target ata-mir168-3p, zma-miR168a-3p |
| OS614 | CACAAATTTCCTGCGTGTTG | RT-qPCR F MLOC_54637.1 target ata-mir166d-5p |
| OS615 | TACCACTTGGTGAACGCAAT | RT-qPCR R MLOC_54637.1 target ata-mir166d-5p |
| OS616 | ATGTGGTCTGGATCGTGTCG | RT-qPCR F MLOC_56778.1 target ath-mir8175 |
| OS617 | CTACAGGCAGCGACCACAA | RT-qPCR R MLOC_56778.1 target ath-mir8175 |
| OS618 | ACTACATCGTCCAAGTGCCG | RT-qPCR F MLOC_39969.1 target gma-mir6300 |
| OS619 | AGATGGTACGGCTGTGAAGC | RT-qPCR R MLOC_39969.1 target gma-mir6300 |
| OS620 | GTTCCAATGGTGCTCCCTGA | RT-qPCR F MLOC_56091.3 target bdi-mir5054 |
| OS621 | GTCACCACGTCGAGGAGG | RT-qPCR R MLOC_56091.3 target bdi-mir5054 |
| OS622 | CGACTCCAGAGCAGGTGAAG | RT-qPCR F MLOC_71386.1 target bdi-mir5054-1 |
| OS623 | TTCATGGAAATGCGGCCTTT | RT-qPCR R MLOC_71386.1 target bdi-mir5054-1 |
| OS626 | GTTTGCAAATCAGGGTCCCC | RT-qPCR F MLOC_63938.2 target bna-mir167d |
| OS627 | ACAAACTCCTGCCATGGGTC | RT-qPCR R MLOC_63938.2 target bna-mir167d |
| OS628 | AATGAAAGGTCCGGACGCAT | RT-qPCR F MLOC_62426.1 target hvu-miR156a/b |
| OS629 | GGACGAGACAAGATGCTTGC | RT-qPCR R MLOC_62426.1 target hvu-miR156a/b |
| OS630 | CCTTCAAGACCGAGCTCTCC | RT-qPCR F MLOC_75555.1 target ppt-mir894 |
| OS631 | CTTCATTCCTCATCCGTGCG | RT-qPCR R MLOC_75555.1 target ppt-mir894 |
| OS632 | TGTGGGAGCTGAACCTTAGT | RT-qPCR F MLOC_77140.1 target ppt-mir894 |
| OS633 | TGAATCACAGCACCACACCA | RT-qPCR R MLOC_77140.1 target ppt-mir894 |
| OS634 | CGTACCCTGCTTGTCGACAT | RT-qPCR F MLOC_21417.1 target hvu-mir5051 |
| OS635 | ATACTCAACAGACGCCTCGC | RT-qPCR R MLOC_21417.1 target hvu-mir5051 |
| OS636 | GACAACTCGAAGACCACCAC | RT-qPCR F MLOC_74285.1 target ata-miR171a-3p |
| OS637 | GATCGACTCTCCCAGCCTC | RT-qPCR R MLOC_74285.1 target ata-miR171a-3p |
| OS638 | AAGAGCAAGGTCACCGTCA | RT-qPCR F MLOC_60369.1 target bdi-miR827-5p |
| OS639 | TAATCTTCCTCCTCGCCCTC | RT-qPCR R MLOC_60369.1 target bdi-miR827-5p |
| OS640 | ACGAAGCATGATTGATCCGC | RT-qPCR F MLOC_19378.1 target bdi-miR827-5p |
| OS641 | CATCAGGGCCATGTCTTTGT | RT-qPCR R MLOC_19378.1 target bdi-miR827-5p |
| OS642 | TTCATTCATGGACCCCGGAT | RT-qPCR F MLOC_79673.4 target hvu-miR171-5p |
| OS643 | GAACGGAGGGAGTACAAGGT | RT-qPCR R MLOC_79673.4 target hvu-miR171-5p |
| OS644 | ACGGAGGGAGAGTGCTAGTA | RT-qPCR F MLOC_52040.2 target ata-miR166c-5p |
| OS645 | TGCTCTCCGTCCATCTCTTC | RT-qPCR R MLOC_52040.2 target ata-miR166c-5p |
| OS646 | GGGGTCAGGCATATCACAGT | RT-qPCR F MLOC_60154.1 target ata-miR166a-5p |
| OS647 | CAACTGCGTACGTCCAATGT | RT-qPCR R MLOC_60154.1 target ata-miR166a-5p |
| OS648 | TGTGGGTGAAGATGTTGATGG | RT-qPCR F MLOC_70788.1 target ata-miR1432-5p, bdi-miR1432 |
| OS649 | AAGCACCACAGAGTCTACGG | RT-qPCR R MLOC_70788.1 target ata-miR1432-5p, bdi-miR1432 |
| OS650 | TCCGTCGACCTGCATACTAC | RT-qPCR F MLOC_70272.1 target ata-miR1432-5p, bdi-miR1432 |
| OS651 | ATGACGAGCGAGATCAGGAG | RT-qPCR R MLOC_70272.1 target ata-miR1432-5p, bdi-miR1432 |
| OS652 | TGGAGGAAGATGATGACGGC | RT-qPCR F MLOC_59888.1 target ata-miR5168-5p |
| OS653 | AAGGAACGGTCTCTCTCTCG | RT-qPCR R MLOC_59888.1 target ata-miR5168-5p |
| OS654 | GAGGAGCACAAGATCGAGGA | RT-qPCR F MLOC_62499.1 target aly-miR396b-5p |
| OS655 | CCTTCATGCCAAGCTTCTCC | RT-qPCR R MLOC_62499.1 target aly-miR396b-5p |
| OS658 | AGCCAACTCTAGCAGCAAGA | RT-qPCR F MLOC_71332.2 target aly-miR159, bdi-miR159a-3p, osa-miR319a-3p.2-3p |
| OS659 | ACTTGAGCCAGCTATTCGGA | RT-qPCR R MLOC_71332.2 target aly-miR159, bdi-miR159a-3p, osa-miR319a-3p.2-3p |
| OS660 | GCGAAGAACCCGTACAAGAG | RT-qPCR F MLOC_55324.1 target hvu-miR159a/b |
| OS661 | CCGCTGCTGTACATTTGGTT | RT-qPCR R MLOC_55324.1 target hvu-miR159a/b |
| OS662 | TCTCCCCAGCATCATCCATC | RT-qPCR F MLOC_22278.1 target ata-miR398f-3p |
| OS663 | AGAAGATGGTGCCCTTGACA | RT-qPCR R MLOC_22278.1 target ata-miR398f-3p |
| OS664 | AGCGTTGTTGTCTTTGGGTG | RT-qPCR F MLOC_65338.1 target hvu-miR6196 |
| OS665 | CTCCAAACTCGGCACCTACT | RT-qPCR R MLOC_65338.1 target hvu-miR6196 |
| OS666 | CGTTGTAGAGTCCATGCAGC | RT-qPCR F MLOC_62884.3 target ata-miR167c-3p |
| OS667 | TTCCTTCCTTCCTCTGCACA | RT-qPCR R MLOC_62884.3 target ata-miR167c-3p |
| OS668 | CGTGTCTGTTGTCTGTAGCG | RT-qPCR F MLOC_12000.1 target ata-miR171c-5p |
| OS669 | ACGCACAGTAGCACATCTCT | RT-qPCR R MLOC_12000.1 target ata-miR171c-5p |
| OS670 | GCTCTGAGAGGTGAAATGGC | RT-qPCR F MLOC_36752.2 target bdi-miR159b-5p.1 |
| OS671 | AACGGAGAAGCCCATCAGAG | RT-qPCR R MLOC_36752.2 target bdi-miR159b-5p.1 |
| OS674 | TCTGGGTGTTTCTGTGAGCA | RT-qPCR F MLOC_74910.1 target aly-miR399b-3p |
| OS675 | CACAAAGACACAGCCAAAACA | RT-qPCR R MLOC_74910.1 target aly-miR399b-3p |
| OS676 | GAAAGGGTTGCTGCAGACAA | RT-qPCR F MLOC_12967.2 target ata-miR166e-5p |
| OS677 | AAATAACTGCTGGCCAAAATCTT | RT-qPCR R MLOC_12967.2 target ata-miR166e-5p |
| OS678 | CAATGAGCTAGCGGTGATGC | RT-qPCR F MLOC_12012.1 target bdi-miR159b-5p.3 |
| OS679 | TGACTCTCCTCAGGTCTCCC | RT-qPCR R MLOC_12012.1 target bdi-miR159b-5p.3 |
| OS680 | CCAGAGCAGGAGGACAAGAA | RT-qPCR F MLOC_69252.1 target bdi-miR164a-3p |
| OS681 | GGCTTGCAGGAGTTGTTCAT | RT-qPCR R MLOC_69252.1 target bdi-miR164a-3p |
| OS682 | CATGCCATACGTAGAAGCTGG | RT-qPCR F MLOC_39634.2 target hvu-miR5049f |
| OS683 | ATGCCAGTCATCCCACCAC | RT-qPCR R MLOC_39634.2 target hvu-miR5049f |
| OS684 | GCATCGCGGTGGTTGAATTT | RT-qPCR F MLOC_5739.1 target ata-miR395a-3p |
| OS685 | GCATCTGCGGTGACCAAAAG | RT-qPCR R MLOC_5739.1 target ata-miR395a-3p |
| OS686 | TTGCATGCCAGTTTTGAGCC | RT-qPCR F MLOC_4408.1 target ata-miR395a-3p |
| OS687 | TGCTGAATGTAGGGCGGATT | RT-qPCR R MLOC_4408.1 target ata-miR395a-3p |
| OS688 | TTTTGCCCTGGACATGTCGT | RT-qPCR F MLOC_21142.1 target ata-miR408-5p |
| OS689 | CCATAGCAAGGGTAGAGCATAAC | RT-qPCR R MLOC_21142.1 target ata-miR408-5p |
| OS690 | CACAGGGAGCAGCTACAACA | RT-qPCR F MLOC_53744.1 target aly-miR164a-5p |
| OS691 | TGTCACCGGGATGTTCATCG | RT-qPCR R MLOC_53744.1 target aly-miR164a-5p |
| OS692 | ATGCCACCATGAACACCTCC | RT-qPCR F MLOC_64240.2 target aly-miR164a-5p |
| OS693 | TCATGCCATTACCGTTGCCT | RT-qPCR R MLOC_64240.2 target aly-miR164a-5p |
| OS694 | CGCTGAGACTTGACCCTGAG | RT-qPCR F MLOC_80410.1 target bdi-miR156h-3p |
| OS695 | CTAGTGTGTCAACGGCCCTC | RT-qPCR R MLOC_80410.1 target bdi-miR156h-3p |
| OS696 | GCATTCGAGACATTGCCACA | RT-qPCR F MLOC_64091.1 target bdi-miR156h-3p |
| OS697 | ACCACACAAATACATCAGCTCAC | RT-qPCR R MLOC_64091.1 target bdi-miR156h-3p |
| OS698 | ATCTGTGTTTGCGTCGGGAT | RT-qPCR F MLOC_4830.1 target ata-miR156c-3p |
| OS699 | TATTTCGACACGCACCCCTC | RT-qPCR R MLOC_4830.1 target ata-miR156c-3p |
| OS726 | CCGCTTGCAGAGACACAATC | RT-qPCR F MLOC_43830.1 HORVU.MOREX.r2.2HG0170380.1 Cly1(=AP2 family), target hvu-miR172b-3p 172-3p, 3'UTR |
| OS727 | TCTGCTGGTAATGGCTGTGG | RT-qPCR R MLOC_43830.1 HORVU.MOREX.r2.2HG0170380.1 Cly1(=AP2 family), target hvu-miR172b-3p 172-3p, 3'UTR |
| OS728 | CTGGTGGCTGCAAATTTAGTCGGC | 5' RACE Gene specific primer for trehalose-6-phosphate synthase MLOC_11773.1 HORVU.MOREX.r2.5HG0396750.1 |
| OS729 | TCCCCTTCAATTCCCTTCCC | RT-qPCR F MLOC_43041.2 AP2 family target hvu-miR172b-3p, 5'UTR HORVU.MOREX.r2.7HG0618740.1 |
| OS730 | GAATCTGCAGGGTGGGTTTG | RT-qPCR R MLOC_43041.2 AP2 family target hvu-miR172b-3p, 5'UTR HORVU.MOREX.r2.7HG0618740.1 |
| target for miRNA827_forward | GCGATTCTTGGGTTGACTGT | RT-qPCR SPX-MFS1 (Syg1/Pho81/XPR1-MAJOR FACILITATED SUPERFAMILY1) [HORVU2Hr1G094690] |
| target for miRNA827_reverse | CATGATTTCAGATGCCACCA | RT-qPCR SPX-MFS1 (Syg1/Pho81/XPR1-MAJOR FACILITATED SUPERFAMILY1) [HORVU2Hr1G094690] |
| APO783 | AAACAAAATGTACGACCTTCATC | F Dwarf14 promoter, control of gDNA presence in cDNA |
| APO784 | TTTTGGTTTCATTACTTATCCCTA | R Dwarf14 promoter, control of gDNA presence in cDNA |
| APO387 | CGTGACGCTGTGTTGCTTGT | RT-qPCR standard F ADP-ribosylation factor 1-like [GenBank: AJ508228.2] |
| APO388 | CCGCATTCATCGCATTAGG | RT-qPCR standard R ADP-ribosylation factor 1-like [GenBank: AJ508228.2] |
| APO391 | CGTATGTGACAGGCAGAAG | F 3'ADP, cDNA quality control |
| APO392 | TGTAAAACCACGGCACAGAA | R 3'ADP, cDNA quality control |
| APO408 | CACGACAGCCATCTTCATCT | F 5'ADP, cDNA quality control |
| APO409 | GCGTGCTCCGCCTGATCT | R 5'ADP, cDNA quality control |
