# Supplementary material for: MicroRNA172b-5p/trehalose-6-phosphate synthase module stimulates trehalose synthesis and microRNA172b-3p/AP2-like module accelerates flowering in barley upon drought stress
Source: Front Plant Sci. 2023 Mar 6;14:1124785. doi: 10.3389/fpls.2023.1124785 (PMC10025483; doi:10.3389/fpls.2023.1124785)
Supplement: Supplementary file 8 [file Table_8.docx]

**Supplementary Table 8.** mRNA targets which expression pattern in drought and/or rehydration is not reversely correlated with cognate miRNA levels. Target mRNA expression was revealed by RT-qPCR, the results are shown as FC of control plants expression. The levels of target mRNAs under the control conditions were assumed to be 1 for up- or -1 for down-regulated targets, and the levels of targets under stress conditions were quantified in relation to this standard. The up- and down-regulated targets were suggested by a two-tailed Student’s *t*-test (***p≤0.001, **p≤0.01, *p≤0.05). Up, down-regulated miRNAs are marked as red and blue, respectively.

| **microRNA** | **microRNA expression change [log2 fc]** | | **target expression [fc]** | | | | target |
| --- | --- | --- | --- | --- | --- | --- | --- |
|  | **drought** | **rewatering** | **drought** | p-value | **rewatering** | p-value |  |
| gma-miR4995 | 2.59 | 2.92 |  |  |  |  | not identified |
| gma-miR6300 | 2.10 | 1.98 | 8.26 | * | 2.19 | * | Phosphate import ATP-binding protein. PstB |
| ptc-miR6478 | 1.45 | 1.12 | 2.69 | ** | 2.19 | ** | Annexin |
| bdi-miR5054 | 1.03 | 1.68 | 1.10 |  | 1.17 |  | RNA-binding protein |
| gma-miR5368 | 2.02 | 1.06 | 5.47 | ** | 1.34 |  | Pheophorbide a oxygenase. chloroplastic |
| osa-miR5072 | 1.38 | 0.69 | -1.11 |  | 1.31 | ** | Cytochrome b-c1 complex subunit 9 |
| bna-miR167d | 1.37 | 0.34 | -1.05 |  | -1.18 | * | Auxin response factor |
| ppt-miR894 | 0.73 | 0.23 | 1.21 | * | -1.01 |  | ATP synthase subunit epsilon. mitochondrial |
|  |  |  | -1.03 |  | 1.15 |  | unknown function |
| hvu-miR5051 | -0.73 | -2.06 | -1.84 | *** | 1.18 |  | Ankyrin repeat protein SKIP35 |
| ata-miR168-3p | -0.96 | -1.21 | -2.34 | *** | -1.94 | ** | Glutamyl-tRNA(Gln) amidotransferase subunit A |
| bdi-miR827-5p | -1.85 | -1.01 | 1.24 |  | -1.03 |  | Guanine nucleotide-binding protein subunit alpha-like protein |
| ata-miR166c-5p | -2.32 | -1.96 | -1.27 |  | -1.32 | *** | Fructose-bisphosphate aldolase |
| ata-miR1432-5p | -2.45 | -2.06 | -7.51 | *** | -1.10 |  | Calmodulin. putative |
|  |  |  | -11.21 | *** | -8.80 | *** | Calmodulin. putative |
|  |  |  | -3.17 | *** | -6.11 | *** | 2-oxoglutarate (2OG) and Fe(II)-dependent oxygenase superfamily protein |
| aly-miR396b-5p | -0.63 | -0.56 | -1.28 | * | 1.07 |  | Nascent polypeptide-associated complex subunit alpha-like protein |
| ata-miR166c-3p | -0.73 | 0.01 | -1.44 |  | 1.24 | ** | Homeobox leucine-zipper protein |
| ata-miR5168-3p | -0.82 | -0.29 | -1.44 |  | 1.24 | ** | Homeobox leucine-zipper protein |
| gma-miR156k | -0.86 | -0.41 | -2.07 | * | -1.04 |  | Squamosa promoter-binding-like protein |
| csi-miR166d | -0.87 | -0.24 | -1.44 |  | 1.24 | ** | Homeobox leucine-zipper protein |
| aqc-miR166c | -0.87 | -0.28 | -1.44 |  | 1.24 | ** | Homeobox leucine-zipper protein |
| bdi-miR159a-3p | -0.90 | 0.09 | -1.16 |  | -1.09 | ** | MYB transcription factor |
| hvu-miR159a/b | -1.12 | -0.30 | not expressed |  | not expressed |  | SPOROCYTELESS-like EAR-containing protein 2 |
| ata-miR166d-5p | -1.17 | -0.06 | -3.06 | *** | -1.29 | ** | Calreticulin |
|  |  |  | -3.15 | *** | -4.43 | *** | Cytochrome P450 |
| hvu-miR6196 | -1.22 | -0.54 | -1.04 |  | 1.90 | ** | Eukaryotic translation initiation factor 3 subunit L |
| osa-miR319a-3p.2-3p | -1.53 | -0.23 | -1.16 |  | -1.09 | ** | MYB transcription factor |
| ata-miR171c-5p | -1.60 | 0.50 | -1.19 |  | 1.21 | * | Protein EFR3 |
| aly-miR166a-5p | -1.60 | -0.86 | -3.06 | *** | -1.29 | ** | Calreticulin |
| osa-miR166e-3p | -1.79 | 0.20 | -1.44 |  | 1.24 | ** | Homeobox leucine-zipper protein |
| bdi-miR159b-5p.1 | -1.93 | -0.87 | -1.41 | * | -1.60 | ** | Mitogen-activated protein kinase |
| aly-miR399b-3p | -1.93 | -0.58 | -2.98 | *** | -3.37 | *** | Ascorbate peroxidase |
| ata-miR166e-5p | -2.03 | -0.56 | 1.23 |  | 2.04 | *** | Trafficking protein particle complex subunit 2 |
| bdi-miR159b-5p.3 | -2.24 | 0.25 | -2.59 | *** | 1.25 |  | PsbP-like protein 1 |
| ata-miR395a-3p | -2.54 | 0.48 | 1.31 |  | 1.47 | *** | MYB family protein |
| ata-miR408-5p | -4.20 | -0.93 | 1.56 |  | 2.26 |  | BSD domain (BTF2-like transcription factors. Synapse-associated proteins and DOS2-like proteins) |
| aly-miR164a-5p | 0.12 | 1.88 | 3.53 | *** | 1.65 | * | NAC domain protein |
| cme-miR156j | 0.79 | 0.87 | -2.07 | * | -1.04 |  | Squamosa promoter-binding-like protein |
| zma-miR168a-3p | 0.03 | -0.86 | -2.34 | *** | -1.94 | ** | Glutamyl-tRNA(Gln) amidotransferase subunit A |
| bdi-miR1432 | -0.30 | -0.95 | -3.17 | *** | -6.11 | *** | 2-oxoglutarate (2OG) and Fe(II)-dependent oxygenase superfamily protein |
|  |  |  | -7.51 | *** | -1.10 |  | Calmodulin. putative |
|  |  |  | -11.21 | *** | -8.80 | *** | Calmodulin. putative |
| tae-miR9773 | -0.64 | -1.04 |  |  |  |  | not identified |
| ata-miR156c-3p | -1.25 | -1.12 | 1.34 |  | -1.11 |  | Squamosa promoter-binding protein. putative |
| bdi-miR408-3p | -0.28 | -1.51 | -2.35 | *** | -1.20 |  | Blue copper protein |
