# Supplementary figures and images for: MicroRNA172b-5p/trehalose-6-phosphate synthase module stimulates trehalose synthesis and microRNA172b-3p/AP2-like module accelerates flowering in barley upon drought stress
Source: Front Plant Sci. 2023 Mar 6;14:1124785. doi: 10.3389/fpls.2023.1124785 (PMC10025483; doi:10.3389/fpls.2023.1124785)

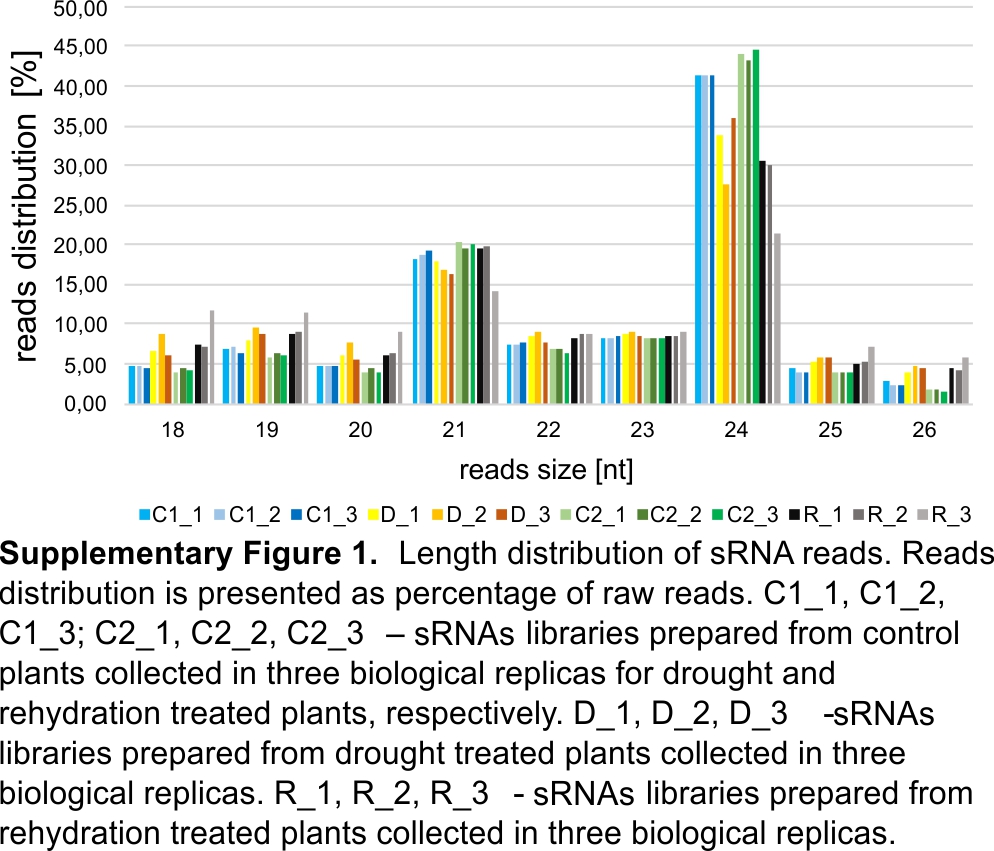

Supplement: Supplementary file 9 [file Image_1.jpeg]

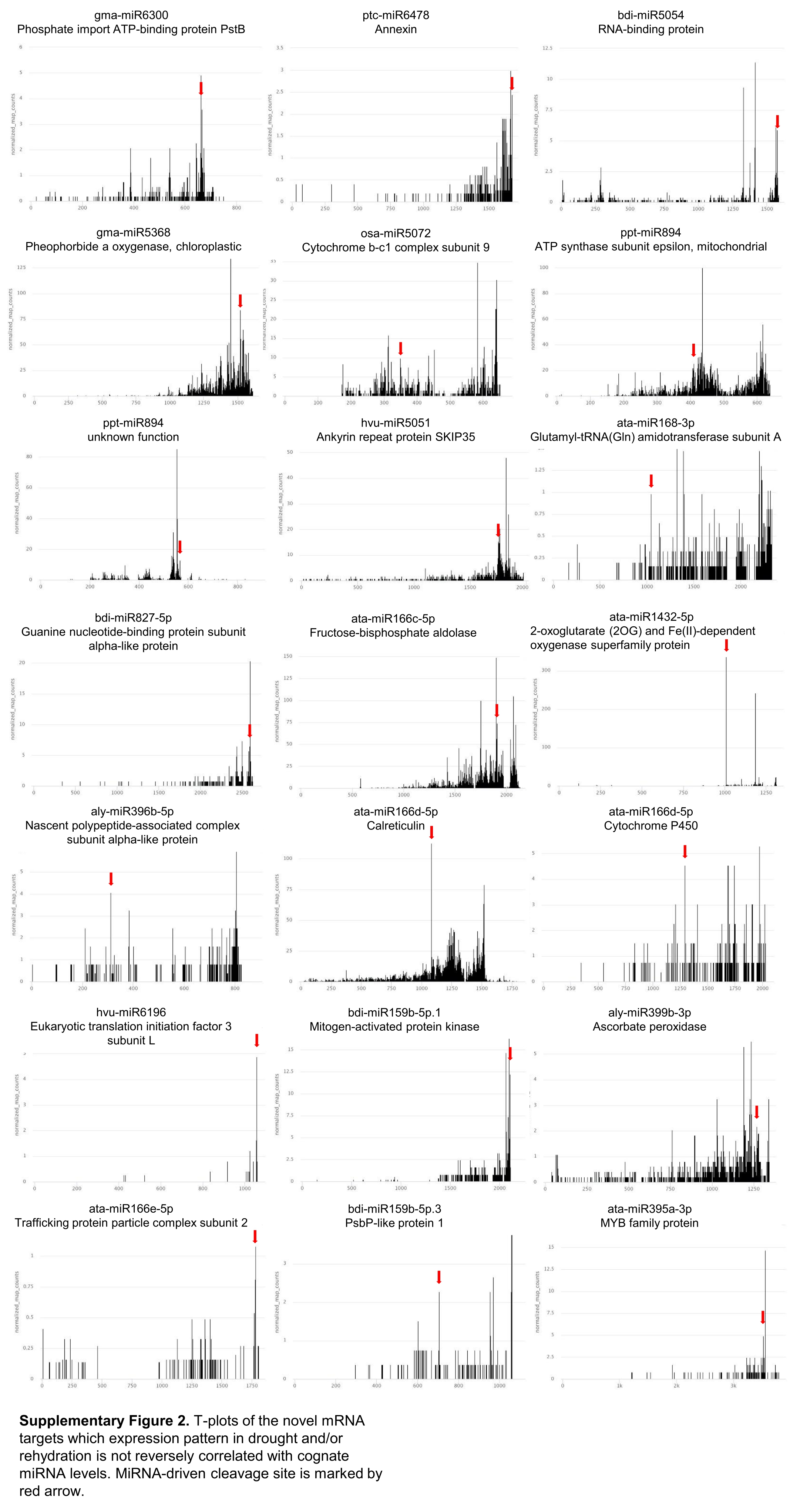

Supplement: Supplementary file 10 [file Image_2.jpeg]
